# Generative psychometrics via AI-GENIE: Automatic item generation and validation with network-integrated evaluation

**DOI:** 10.3758/s13428-026-03082-1

**Published:** 2026-07-01

**Authors:** Lara L. Russell-Lasalandra, Alexander P. Christensen, Hudson Golino

**Affiliations:** 1 University of Virginia, Charlottesville, VA 22904 USA; 2 Vanderbilt University, Nashville, TN 37240 USA

**Keywords:** Psychological scale development, Artificial intelligence, Large language models, Network psychometrics, Exploratory graph analysis, Unique variable analysis, Generative psychometrics, AI-GENIE

## Abstract

The rapid advancement of artificial intelligence (AI), particularly large language models (LLMs), has introduced powerful tools for various research domains, including psychological scale development. This study presents a methodology for efficiently generating and selecting high-quality, non-redundant items for psychological assessments using LLMs and network psychometrics. Our approach, termed Automatic Item Generation and Validation with Network-Integrated Evaluation (AI-GENIE), reduces reliance on expert intervention by integrating generative AI with the latest network psychometric techniques. The efficacy of AI-GENIE was evaluated through Monte Carlo simulations using the Mixtral, Gemma 2, Llama 3, GPT-3.5, and GPT-4o models to generate item pools that mimic Big Five personality assessments. Additionally, items from AI-GENIE were empirically tested with five nationally representative U.S. samples ($N = 4{,}964$ total), demonstrating that AI-GENIE-generated scales achieve structural validity—that is, evidence based on internal structure (dimensionality and item stability)—comparable to traditional expert-developed measures. The results demonstrated improvements in item selection efficiency, with overall average increases of 8.68–20.03 in normalized mutual information in the final item pool across all models. We also present a simulation study on the emerging construct of AI anxiety to demonstrate AI-GENIE’s utility for underrepresented constructs. Results from newly released models (DeepSeek, GPT-OSS 20B, GPT-OSS 120B) are presented in the Appendix. The findings suggest that AI-GENIE can streamline the scale development and structural validation process.

## Introduction

The development of psychological scales is a resource-intensive and challenging process, particularly during the crucial stages of item generation and structural validation. Traditional methods require extensive human intervention, making the process time-consuming and costly. In response to these challenges, this paper introduces *Automatic Item Generation and Validation with Network-Integrated Evaluation* (AI-GENIE), a novel methodology for item development and structural validation in silico, leveraging the capabilities of large language models (LLMs) and advanced network psychometric techniques. This new approach streamlines, simplifies, and makes scale development more cost-effective while maintaining the quality and structural validity of the generated items. AI-GENIE begins by prompting an LLM to generate an initial pool of items; these items are then evaluated and refined using a multi-step psychometric pipeline that includes redundancy detection and stability-based filtering. The present AI-GENIE methodology has been integrated into an R package called AIGENIE available on the R Universe. For more details on the package and its usage, see Russell-Lasalandra and Golino (2026).

Consider the task of measuring the conscientiousness trait from the popular Big Five model of personality (John, 1999). Traditionally, researchers would manually craft items like “I keep my work organized” or “I meet deadlines reliably” based on their theoretical understanding and iterative empirical testing. Then, researchers have to repeat the process for the four other personality traits. This painstaking, construct-by-construct development is not an edge case. It is the norm. AI-GENIE partially automates this process by prompting LLMs to generate dozens of candidate items for conscientiousness, then evaluates and filters these using network psychometrics to arrive at a concise, high-quality scale.

Scale development is fundamental to psychological measurement, and the number of researchers developing scales has increased in recent years. A search of PsycTests reveals that 761 scales were published to the site in the first eight months of 2023 alone (this figure is much higher than when Götz et al. (2023) cited the same figure from 2022). Despite its ubiquity, scale development is a challenging, costly, and time-intensive undertaking (Boateng et al., 2018). One especially arduous, yet critical, step in the scale development process is item generation. This step can be difficult for several reasons: (1) major deficiencies in item quality cannot be corrected after items are written, (2) the initial pool should be so comprehensive that some items are ultimately shown to be unrelated to the target construct, and (3) the initial pool should include up to five times as many items as desired in the final scale (Clark & Watson, 2016; Schinka & Velicer, 2012). Given the challenges of item generation and the sheer writing power of modern LLMs, researchers have begun to test the efficacy of AI-generated items for psychological assessments (Hommel et al., 2022; Kjell & Kjell, 2023; Maertens et al., 2024). These efforts reflect a broad and rapidly emerging research program, which we term *generative psychometrics*: the use of generative AI and text embeddings to author, refine, and evaluate the building blocks of psychological measurement.

Recent advances in LLMs, with billions of parameters and vast pre-trained knowledge (Brown et al., 2020; Raffel et al., 2023), can generate expert-quality text without task-specific retraining (Carlini et al., 2021). To our knowledge, the present paper is the first to introduce the term generative psychometrics in this modern sense. Since then, a rapidly growing literature has begun to populate the area, reflecting a broader shift toward treating language as a scalable behavioral trace for psychological science (Brickman et al., 2025; Debelak et al., 2025; Feuerriegel et al., 2025; Galatzer-Levy et al., 2026; Jackson et al., 2022; Kjell &, 2024). As Garrido et al. (2025) points out, LLMs are now embedded in item-generation workflows that expand, refine, translate, and cross-culturally adapt item pools, typically under structured human oversight or multi-agent quality-control pipelines (Fyffe & Lee, 2024; Grobelny &, 2025; Hernandez & Nie, 2023; Kopka et al., 2025; Kowal &, 2025; Perrin &, 2025), with mounting evidence that machine-authored items can match or even exceed human-authored ones in model fit (Hommel et al., 2022). Text embeddings, which map item stems and construct labels into dense high-dimensional vectors whose geometry encodes graded semantic relations (Fang & Oberski, 2022; Hussain et al., 2024), now support scale abbreviation without response data, nomological mapping across hundreds of thousands of indicators, pre-data-collection diagnostics of content and construct validity, and the clarification of construct relationships and psychological taxonomies (Kilmen, 2025; Larsen &, 2025; Wulff, 2023, 2025a, b). Relatedly, both embeddings and language models have been shown to approximate psychometric parameters that traditionally required empirical response data (Fyffe & Lee, 2024; Guenole et al., 2024). In parallel, transformer-based models are used as automated raters and scoring engines for many constructs derived from open-ended text (Bunt & Gillespie, 2025; Hommel, 2023; Maharjan &, 2025), and LLMs can be prompted to emulate specific respondent profiles to produce synthetic pseudo-response data analyzable with conventional psychometric models (Cipriani & Grassini, 2025; Niszczota &, 2025). Complementary methodological work has examined prompt engineering for item generation (Russell-Lasalandra, 2026) and the behavior of dimensionality estimators applied to embedding-based similarity matrices (Garrido & Russell-Lasalandra, 2025; Golino, 2025).

Yet for all of this progress, a critical gap remains. Within this emerging field, the central challenge is not merely generating items but efficiently selecting and structurally validating those that are non-redundant and faithful to the intended construct. Götz et al. (2023), for example, used GPT 2 (Radford et al., 2019) to draft items for a Big Five inventory and a novel “wanderlust” construct. While the resulting scales met standard quality benchmarks, the process of identifying viable items was resource-intensive: a committee of four expert psychologists independently reviewed thousands of generated items, ultimately retaining fewer than one hundred (a yield below 0.03%). Although the best AI-authored items were of expert quality, identifying them required nearly as much effort as traditional human authorship (DeVellis, 2017). Despite several recent propositions to develop and select items via LLMs (see Laverghetta et al. (2024), our methodology is, to our knowledge, the first to generate, assess, and structurally validate the quality of AI-generated items for psychometric scales within a single integrated pipeline.

Critically, while we demonstrate AI-GENIE using AI-generated items, the pipeline itself is deterministic and source-agnostic: given the same item pool and embedding model, AI-GENIE will produce identical results regardless of whether items were authored by humans, AI, or both. This reproducibility and flexibility mean researchers can apply our psychometric refinement methodology to any item pool that is sufficiently large, making AI-GENIE a general-purpose tool for systematic item selection that extends beyond AI-based generation. Having established what AI-GENIE does, it is equally important to be precise about what it is designed to do.

Throughout this paper, we use the term validation to refer specifically to what Christensen et al. (2020) call the “structural phase” of validation, i.e., assessing item redundancy, dimensionality, and internal consistency, which corresponds to evidence based on internal structure as outlined in the Standards for Educational and Psychological Testing (Aera et al., 1999; Rios & Wells, 2014). This scope does not extend to other sources of validity evidence (e.g., convergent, discriminant, or criterion-related). However, AI-GENIE *does* systematically check the structural validity of candidate items entirely *automatically.*

To carry out this structural validation, AI-GENIE uses exploratory graph analysis (*EGA*; Golino and Epskamp, 2017) and related network-based approaches rather than principal component analysis (PCA). Structural analysis of embedding-based similarity matrices in the literature has been dominated by principal component analysis (Bhandari & Pardos, 2024; Cutler, 2023; Huang &, 2025; Jung, 2025; Linden et al., 2025; Milano &, 2025), reflecting a long historical tradition of PCA as the default tool for deriving multilevel structure from large item pools (Ashton & Goldberg, 2004; Goldberg, 2013; Saucier, 2020). Recent simulation work, however, shows that PCA severely overestimates dimensionality when applied to embedding-based similarity matrices (mean bias error = 15.06; accuracy = 0%), whereas EGA achieves *markedly* better dimensional recovery (mean bias error = 0; accuracy = 95.6%) after item filtering, removing redundancy and unstable items (Garrido & Russell-Lasalandra, 2025).

The remainder of the paper is organized as follows. We first provide a high-level overview of the techniques underlying AI-GENIE, including LLM-based item generation, embeddings, and the Network-Integrated Evaluation pipeline (UVA, EGA, and bootEGA). The Methods section details the Monte Carlo simulation design, prompt engineering strategy, and the AI-GENIE pipeline. The Results section reports item yield, redundancy reduction, dimensional recovery, and stability across models and temperatures, followed by an empirical validation with five nationally representative U.S. samples and a focused simulation on AI Anxiety as an underrepresented construct. We close with a discussion of implications, limitations, and future directions for generative psychometrics. Results for newly released models (DeepSeek, GPT-OSS 20B, GPT-OSS 120B) are reported in the Appendix.

### Overview of techniques

Before diving into the specific components of AI-GENIE, we provide a high-level overview of its techniques. All LLMs are advanced AI systems built to process and generate human language in context (Hadi et al., 2023). Fundamental to LLMs’ ability to represent the contextual meaning of human language are their embeddings, high-dimensional numeric vector representations of text that capture semantic meanings, relationships, and, importantly, the context within the text data (Vaswani et al., 2017). We use OpenAI’s text-embedding-3-small model to embed the text data (Openai, 2024a), enabling relationships between items to be represented numerically.

In the AI-GENIE pipeline, item generation via generative models is followed by an item-relation phase via LLM embeddings. The specific models tested, their selection rationale, and the role of the Groq Cloud API in low-latency inference are detailed in the Methods section below.

A model’s *temperature* is a decoding hyperparameter that rescales the next-token probability distribution, and is often described as a “creativity” knob, though the relationship to creativity is not always direct (Peeperkorn et al., 2024). Increasing temperature flattens the distribution, making atypical tokens more likely and responses more diverse; decreasing temperature sharpens it, yielding more predictable outputs. The impact of temperature on output quality is task-dependent: one study found little effect on problem-solving performance (Renze, 2024), whereas another reported measurable changes in code-generation quality with temperature tuning (Zhu et al., 223). Accordingly, temperature was varied to assess its influence in this application.

After generating items and obtaining their embeddings, item selection and structural validation begins with a set of techniques we are calling network-integrated evaluation, which uses methods developed within the EGA framework. The pipeline follows what has become the standard workflow of this framework (e.g., Krugel and Christensen, 2025). The process starts by employing unique variable analysis (*UVA*; Christensen et al., 2023) to automatically identify and eliminate redundant items in the item pool. Next, EGA is applied to estimate the dimensionality structure of the remaining items, using network psychometrics and community detection algorithms (e.g., Walktrap; (Pons, 2005)). In the final stage of AI-GENIE, bootstrap EGA (*bootEGA*; Christensen and Golino, 2021) is employed to evaluate the stability of the items in each dimension and remove items that do not contribute to the overall stability of each dimension. This final part is critical because it is used to determine an item’s quality in measuring a stable construct.

The present research tested the efficacy of the AI-GENIE methodology using a Monte Carlo simulation under which the type and temperature of the selected language models were tested. Each of the five models was tested under three temperature settings (0.5, 1, and 1.5) for each of the Big Five personality traits, resulting in a total of 75 experimental conditions. Inferencing (or text generation) on the models was repeated until 100 samples of at least 60 unique items were collected for each of the 75 conditions. The diversity, consistency, and quality of the items produced could be assessed by condition, allowing for the exploration of how different model architectures and temperature settings influence item generation outcomes.

## Methods

### Simulation design, item generation, and prompt engineering techniques

Five large language models were used to generate items for the Big Five traits: agreeableness, conscientiousness, extraversion, neuroticism, and openness to experience (John, 1999). Three of these models, Gemma 2 (Team et al., 2024), Llama 3 (Meta, 2024), and Mixtral 8x7b (Jiang et al., 2024), are open-source models available on the Groq API (groq, 2024). The other two models, GPT-3.5 (Openai, 2024b) and GPT-4o (Openai, 2024c), come from OpenAI.

Results from an additional simulation that includes DeepSeek (Deepseek et al., 2024) and OpenAI’s open-source GPT OSS 20b and 120b (Openai, 2025), released after our main simulation was completed, are reported in the Appendix to illustrate AI-GENIE’s robustness across the evolving model ecosystem.

Each of the five models was given the same few-shot prompt that asked the model to generate eight novel items for a given Big Five personality trait. We kept inferencing the model until a sample of at least 60 unique items for a given trait was obtained. The temperature of the model was either set to low (0.5), medium (1; default), or high (1.5). One hundred samples of at least 60 items were collected for each combination of item type (trait), model, and temperature setting. Thus, a total of 7500 samples of about 60 items were collected across the entire Monte Carlo simulation.

Prompt quality plays a significant role in the consistency and viability of LLM output (Jin et al., 2022; White et al., 2023). The current paper used four established prompt engineering techniques to elicit high-quality items: (1) clear and precise prompting, providing a detailed task description and output format; (2) few-shot prompting, supplying examples of desirable output to improve consistency (Brown et al., 2020; 3) role prompting, assigning the model the identity of *“an expert psychometrician and test developer specializing in personality assessment”* (Zheng & Pei, 2023); and (4) adaptive prompting, instructing the model not to repeat items already generated (Lightman et al., 2023). A detailed examination of how these prompt engineering strategies shape item quality within the AI-GENIE framework is provided in Russell-Lasalandra and Golino (2026).

As part of the few-shot prompting approach, the model was given a list of trait attributes to ensure that generated items targeted several aspects of each Big Five trait. The attributes for each trait were:

1. *Openness*: creative, perceptual, curious, and philosophical
2. *Conscientiousness*: organized, responsible, disciplined, and prudent
3. *Neuroticism*: anxious, depressed, insecure, and emotional
4. *Agreeableness*: cooperative, compassionate, trustworthy, and humble
5. *Extraversion*: friendly, positive, assertive, and energetic

The model was prompted to generate two items per attribute per iteration. Since LLMs are limited in the amount of text they can produce in a single generation, the prompt was presented multiple times until a minimum of 60 non-duplicate items were collected per sample. The adaptive prompting approach mitigated repetition by providing the model with a cumulative list of items already generated. A total of nearly half a million items were generated across all conditions.^1^ The average item count per sample varied minimally between experimental conditions (see Table 7 in the Appendix), so sample size should have a negligible impact on the observed differences in model performance.

### Automatic item generation and validation via network-integrated evaluation (AI-GENIE)

**Fig. 1:**
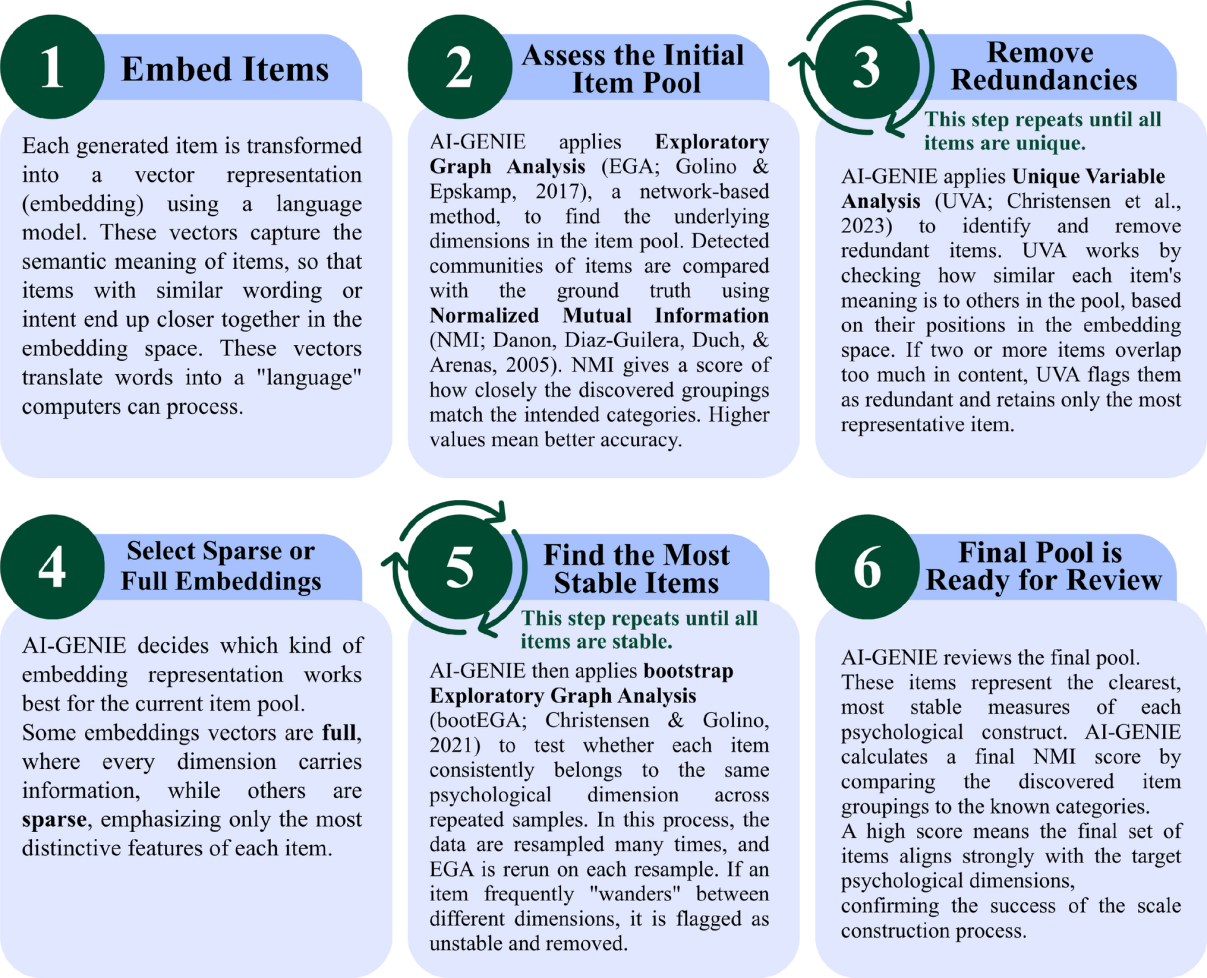
An illustration of the six steps of AI-GENIE with descriptions of each step

The proposed AI-GENIE methodology can use LLMs to generate items, but the core, structural validation portion of the method consists of the following six steps (Fig. 1). Importantly, because the pipeline operates on embedding matrices rather than person-level response data, the data structure differs from traditional psychometric applications: embedding dimensions serve as rows (observations), and items serve as columns (variables). Recent simulation work has demonstrated that EGA and its associated network filtering procedures (UVA, bootEGA) recover dimensional structure from embedding-based similarity matrices with high accuracy, outperforming traditional approaches such as PCA (Garrido & Russell-Lasalandra, 2025).

#### Step 1: Embed the items

The first step of the AI-GENIE is to embed the items. The items were embedded using OpenAI’s *text-embedding-3-small* model (Openai, 2024a), a process facilitated by the {reticulate} package in R [version 1.39.0; Ushey et al. (2023)]. OpenAI’s model, *text-embedding-3-small*, offers significant improvements over its predecessor, *text-embedding-ada-002*, in terms of both performance and cost (Openai, 2024a). Because embeddings translate the semantic meaning of an item into a numeric vector situated within a high-dimensional space (in our case, 1536 dimensions), the dimensions are treated as *“observations”* or rows in a data frame, whereas the items themselves are considered the *“variables”* or columns.

#### Step 2: Perform initial EGA

This phase of AI-GENIE establishes a baseline EGA community detection performance to match or improve upon after item reduction. Community detection with EGA requires two broad steps: (1) constructing a network and (2) finding clusters of nodes within the network. For the second step, the Walktrap (Pons, 2005) community detection algorithm, an algorithm that works by randomly “walking” along the edges of the network to find pockets of particularly interconnected nodes, was used to identify the item clusters. The Walktrap algorithm was selected over others since it has shown superior performance in simulation studies on psychological data generated from factor models (Christensen et al., 2024; Golino et al., 2020), though these simulations used person-level response data rather than embedding matrices. Nevertheless, the Walktrap algorithm’s reliance on random walks to detect densely connected clusters is well suited to embedding-derived networks, and recent evidence confirms its effectiveness in this context (Garrido et al., 2025). For the first step, the efficacy of two network-building or estimation approaches was tested: Triangulated maximally filtered graph (TMFG; Massara et al., 2016) and extended Bayesian information criterion glasso (Foygel and Drton 2010; EBICglasso; Friedman et al., 2008).

TMFG is a greedy algorithm that iteratively builds a maximally filtered network by adding nodes one at a time, maximizing the absolute sum of correlations at each step while keeping the number of edges minimal (Massara et al., 2016). Prior research has shown that TMFG performs well with text data, being more robust for variables with skewed distributions than EBICglasso (Golino et al., 2020). EBICglasso (Epskamp, 2018; Foygel, 2010) takes a complementary approach, estimating a sparse Gaussian graphical model via the glasso method (Friedman et al., 2008) and selecting the optimal sparsity level using the extended Bayesian information criterion. The resulting network retains only the conditional dependencies between items, producing a sparser and more interpretable structure, at greater computational cost.

Once the network is built and the communities have been found, the quality of the EGA fit can be assessed. Typically, fit is evaluated using the Total Entropy Fit Index (TEFI; Golino et al., 2021; Jamison et al., 2021), where lower values indicate a more organized dimensionality structure. However, given that each item was generated from an attribute-specific prompt (e.g., items targeting neuroticism were generated separately for the anxious, depressed, insecure, and emotional attributes), we can define a prompt-based community assignment for each item at the attribute level. We assess the accuracy of the EGA model by comparing its detected communities against these attribute-level assignments using the *normalized mutual information* (NMI; Danon et al., 2005) index, an information-theoretic measure. That is, if an item generated for the “anxious” attribute of neuroticism is placed by EGA into the same community as other “anxious” items, this constitutes a match. Importantly, NMI in the AI-GENIE pipeline is computed at this attribute level, a finer-grained test than simply recovering the five broad traits, which makes high NMI values more difficult to achieve. NMI ranges from 0 (completely dissimilar) to 1 (identical). The closer the dimensionality structure obtained from EGA is to the known communities, the higher the NMI. NMI can be used as a classification matching algorithm, where scores closer to 100 (in a scale from 1 to 100, by multiplying the NMI value by 100) indicate better community detection.

Although NMI is widely used, it is known to be sensitive to the number and size of clusters (Jerdee et al., 2023; Vinh et al., 2010) and may introduce bias through symmetric normalization. To ensure robustness, we also computed adjusted mutual information (AMI; Vinh et al., 2010) and the adjusted Rand index (ARI; Hubert and Arabie, 1985), both of which correct for chance agreement. These results are presented in Tables 9 and 8 of the Appendix.

High NMI is not a foregone conclusion in this pipeline. Although items are generated from trait-specific prompts, the resulting embeddings can be semantically ambiguous. For example, an item generated for the “disciplined” attribute of conscientiousness (e.g., *“I double-check my work before submitting it”*) may embed closer to “responsible” attribute items due to shared anxiety-related connotations. In practice, pre-reduction NMI values varied considerably across conditions, falling as low as 51 in some cases (see Results). This variation demonstrates that EGA does not trivially recover the intended structure from embedding data, and that the improvement in NMI after applying the AI-GENIE pipeline reflects meaningful refinement of the item pool.

In this step of the pipeline, an initial EGA network was built using all items in the item pool. The quality of community detection was assessed using NMI, which was calculated using the *igraph* package in R (version 1.5.1; Csárdi et al., 2024). This step and all subsequent steps are performed using the *EGAnet* package in R (version 2.0.6; Golino and Christensen, 2023).

#### Step 3: Run UVA until all items are unique

The next step in AI-GENIE reduces redundancy in the item pool using unique variable analysis (UVA; Christensen et al., 2023). UVA is a network psychometrics method that detects local dependence (redundancy) among pairs or sets of variables using Gaussian graphical models and a graph theory measure known as weighted topological overlap (wTO; Zhang and Horvath, 2005). wTO quantifies the extent of similarity between pairs of variables (or, in our case, items) in a network based on their shared connections or relationships with other variables in the network. Pairwise relationships are considered redundant if their wTO values fall above a given cut-off value. This process for identifying redundancies works since a group of items that share an almost identical meaning will also have a very similar embedding vector, which means these redundant items will share many of the same connections to other items in the network. Only one item per redundant pair/set is retained; that item is the one whose wTO with respect to all other items in the pool is the lowest. That is, the item that is most dissimilar to all other items in the pool is kept and the other item(s) in the pair/set get discarded, strategically shrinking the item pool. However, redundancy detection may not yet be complete. After this first sweep, UVA runs again on the redundancy-reduced pool. If the second sweep of UVA identifies more redundancies, the appropriate items are discarded and UVA runs once again. This process continues until UVA identifies no further redundancies.

Simulations on person-level response data have shown that a standard cut-off value of 0.25 is appropriate for a majority of applications (Christensen et al., 2023). However, initial experimentation revealed that this standard value was too conservative, as UVA failed to identify items that were worded almost identically. We found that a cut-off value of 0.20 could better capture more of the highly redundant items. While this lowered cut-off value can lead to a misidentified pair in very rare instances, this lowered value is generally able to capture many more redundancies without erroneously discarding many non-redundant items.

**Fig. 2:**
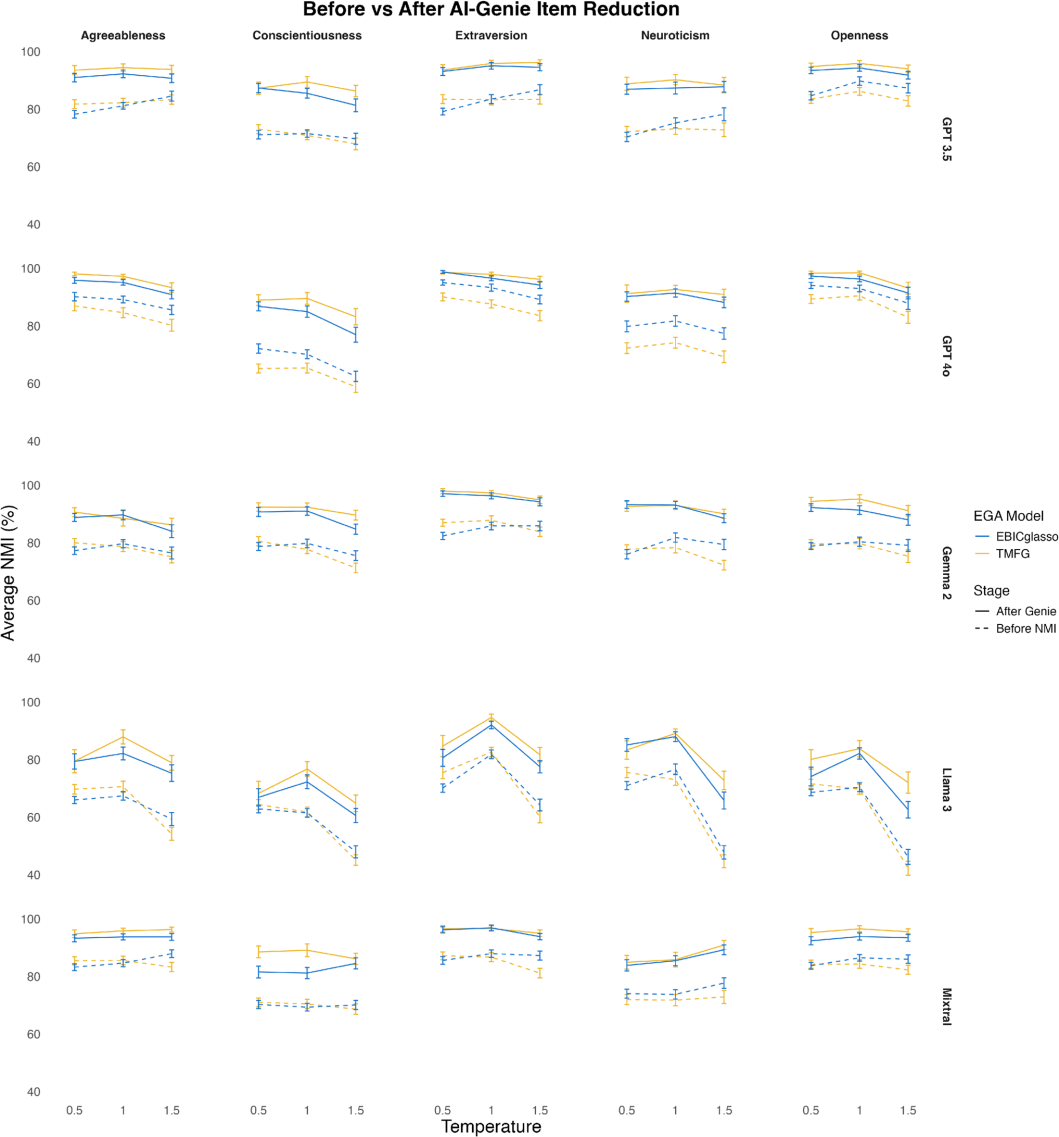
The relationship between the initial NMI (*dashed*), final NMI (*solid*), model temperature, LLM model, EGA model, and item type

**Table 1:** Mean improvement in NMI after AI-GENIE implementation

	AGR		CONSC		EXTR		NEUR		OPEN		Grand Mean
	TMFG	GLASSO	TMFG	GLASSO	TMFG	GLASSO	TMFG	GLASSO	TMFG	GLASSO	
GPT-3.5											
Temp 0.5	11.83	12.78	14.24	16.26	10.11	13.93	16.61	16.55	11.31	8.77	**13.24**
Temp 1	12.2	11.12	18.55	14	12.62	11.58	17.07	12.12	9.7	4.56	**12.35**
Temp 1.5	10.46	6.2	18.38	11.62	12.81	7.82	15.52	9.59	11.16	4.5	**10.81**
Mean	**11.49**	**10.03**	**17.06**	**13.96**	**11.84**	**11.11**	**16.4**	**12.75**	**10.73**	**5.94**	
GPT-4o											
Temp 0.5	11.11	5.67	23.75	14.68	8.55	3.69	18.96	10.39	9.01	3.21	**10.9**
Temp 1	12.63	5.95	24.21	14.85	10.31	3.27	18.49	9.71	8.04	3.31	**11.08**
Temp 1.5	13.08	5.32	24.35	14.48	12.64	5.06	21.67	10.8	10.16	3.55	**12.11**
Mean	**12.27**	**5.65**	**24.1**	**14.67**	**10.5**	**4.01**	**19.71**	**10.3**	**9.07**	**3.36**	
Gemma 2											
Temp 0.5	10.62	11.54	11.84	12.01	10.97	14.7	14.91	17.29	14.73	13.39	**13.2**
Temp 1	9.83	10.02	14.69	11.24	9.58	10.46	14.69	11.31	15.53	11.02	**11.84**
Temp 1.5	11.14	7.55	18.34	9.21	11.11	8.38	17.84	9.13	15.86	8.87	**11.74**
Mean	**10.53**	**9.7**	**14.96**	**10.82**	**10.56**	**11.18**	**15.82**	**12.58**	**15.37**	**11.09**	
Llama 3											
Temp 0.5	9.73	13.41	3.92	4.03	9.2	10.52	7.82	14.12	8.48	5.52	**8.68**
Temp 1	17.34	14.77	14.96	10.83	12.01	10.22	16.07	11.34	14.1	11.76	**13.34**
Temp 1.5	24.74	15.98	19.76	12.63	21.55	13.32	28.12	18.02	29.78	16.44	**20.03**
Mean	**17.27**	**14.72**	**12.88**	**9.16**	**14.25**	**11.36**	**17.34**	**14.49**	**17.45**	**11.24**	
Mixtral											
Temp 0.5	9.37	10.04	17.53	11.34	9.38	10.64	12.97	9.86	10.96	8.67	**11.08**
Temp 1	10.3	9.12	18.68	11.87	9.97	8.96	14.05	11.7	12.24	7.39	**11.43**
Temp 1.5	13.03	5.83	17.48	14.46	13.85	6.59	18.06	11.57	13.24	7.46	**12.16**
Mean	**10.9**	**8.33**	**17.9**	**12.56**	**11.07**	**8.73**	**15.03**	**11.04**	**12.15**	**7.84**	
Grand mean	**12.49**	**9.69**	**17.38**	**12.23**	**11.64**	**9.28**	**16.86**	**12.23**	**12.95**	**7.89**	

Bold in the table represent grand means

In addition to lowering the wTO cut-off value, using a sparse embedding matrix (where most vector components are zeros, retaining only the most informative dimensions) improved redundancy detection. Noise in full embedding matrices can obscure the similarity between redundant items, reducing their topological overlap (Derby et al., 2018; Guillot & Prouteau, 2023; Mandikal, 2024). Using sparse embeddings mitigates this issue and increases the number of detectable redundancies.

#### Step 4: Determine embedding type to use

Similarly, depending on the makeup and size of the item pool, the subsequent bootEGA step may perform better with the sparse embeddings. Thus, to determine which embedding type to use (either sparse or full), the NMI of the EGA network constructed using the sparse embeddings is compared to that of the full embeddings. The embedding type whose EGA network produces the highest NMI is selected for use in all subsequent steps of the pipeline.

#### Step 5: Perform bootEGA until all items are stable

The penultimate step of the pipeline employs bootEGA, which estimates the stability of EGA (and, by extension, the item stability) across several bootstrapped samples (Christensen & Golino, 2021). An item’s quality can be inferred from the item’s stability, which is the tendency of an item to stay in the same cluster across multiple bootstrapped samples. Items that are weakly related to the other items in their typical community will wander from community to community in some of the sub-samples of the data set. Conversely, items that are strongly related to a majority of items in their community will almost always cluster in the same community, regardless of the sub-sample. For instance, the item *“I make myself known at parties.”* (an item that was supposed to reflect the “assertive” attribute of extraversion) could occasionally cluster with items related to the “friendly” attribute in certain bootstrapped subsamples. Its instability flagged it as weakly associated with its intended trait, prompting its removal in a later sweep. Further, minor dimensions, which form when there are pockets of highly related items within the major dimensions, can reduce item stability and can also hinder the recovery of the desired (known) communities (Krugel, 2025).

The bootEGA procedure used in this study used 100 sub-samples that were resampled from the dataset and had EGA applied to each. Items with a stability less than 0.75 (or that clustered in the same community as the original communities in fewer than 75 of the sub-samples) were considered unstable (Christensen & Golino, 2021). These unstable items are removed from the item pool. Then, bootEGA runs again for the remaining items. If all items are stable after the second round, the pipeline completes, and the finished item pool is ready for review. Otherwise, the unstable items are removed again, and the process continues. Typically, this step requires 2–3 iterations of bootEGA to identify the most stable items for the final item pool. A more conservative (0.70) and more liberal (0.80) item stability cut-off value was also tested, but these values led to the removal of topical items or the inclusion of lower-quality items, respectively.

Although the procedure appears to work toward a simple structure, adequate stability can be achieved with complex constructs. The procedure primarily works toward a *stable* structure, which tends to be more simple structure because multidimensional items often cause stability issues (e.g., equivalent stability in two or more dimensions).

#### Step 6: Complete. Review final item pool and find final NMI

After all instabilities have been removed, item reduction is complete, and the final item pool is ready for review. A final EGA network is built for these remaining items, and the NMI is calculated based on the communities detected in this network. This final NMI should be roughly the same as or exceed the initial NMI from Step 1.

## Results

We tested AI-GENIE using a Monte Carlo simulation study under several conditions. Our study modulated both model type (Gemma 2, Llama 3, Mixtral, GPT-3.5, GPT-4o) and temperature setting (0.50, 1.00, 1.50). One hundred samples of about 60 items each were generated for all 75 combinations of item type (i.e., Big Five personality traits), model type, and temperature.

### General results

As shown in Fig. 2 and Table 1, the average final NMI is an improvement over the average initial NMI for all conditions. The two EGA models used, TMFG and EBICglasso, performed comparably, though TMFG generally yielded slightly better results than EBICglasso for some conditions. This result is consistent with previous findings on TMFG in the context of text data (Golino et al., 2020). The highest and second-highest-temperature Llama 3 models showed the largest average improvements of 20.03 and 13.34 NMI, respectively, across all trait types, EGA models, and temperatures. GPT-3.5’s lowest-temperature model showed an overall average change in NMI of 13.24, and Gemma 2’s lowest-temperature model showed 13.20. Llama 3’s 0.5 temperature model showed the smallest improvement of 8.68, though even this model showed consistent gains across all Big Five personality traits.

Across nearly all conditions, AMI and ARI closely tracked NMI, with only minor deviations on average. One notable exception was Llama 3 at both low and high-temperature settings, where AMI and ARI values were approximately ten points lower than NMI on average. Even in this case, the qualitative conclusions remained unchanged: the detected communities were consistent across all three metrics. These results suggest that while NMI has known limitations, in this application it provides a consistent summary of partition alignment, particularly when supported by convergent evidence from AMI and ARI (Tables 9 and 8).

### Steps 1 and 2: Embed items and initial NMI

Once embeddings were generated, the “initial NMI” was found using the entire item pool. The NMI was calculated by comparing each item’s prompt-based attribute assignment with its empirical community identified by EGA. The highest NMI value across the step size parameters was recorded as the “initial NMI” that subsequent steps should preserve or improve upon (Table 2).

**Table 2:** Mean NMI before AI-GENIE implementation

	AGR		CONSC		EXTR		NEUR		OPEN		Grand Mean
	TMFG	GLASSO	TMFG	GLASSO	TMFG	GLASSO	TMFG	GLASSO	TMFG	GLASSO	
GPT-3.5											
Temp 0.5	81.75	78.23	73.06	71.1	83.51	79.19	72.2	70.39	83.53	84.69	**77.76**
Temp 1	82.27	81.18	70.93	71.53	83.29	83.52	73.17	75.26	86.22	89.79	**79.72**
Temp 1.5	83.35	84.55	67.93	69.72	83.45	86.76	72.85	78.21	82.86	87.34	**79.7**
Mean	**82.46**	**81.32**	**70.64**	**70.78**	**83.42**	**83.16**	**72.74**	**74.62**	**84.2**	**87.27**	
GPT-4o											
Temp 0.5	87	90.23	65.26	72.15	90.12	95.12	72.32	79.87	89.4	94.14	**83.56**
Temp 1	84.65	89.22	65.4	70.18	87.68	93.36	74.21	81.78	90.49	93.05	**83**
Temp 1.5	80.28	85.62	58.89	62.5	83.6	89.21	69.31	77.41	83.05	87.94	**77.78**
Mean	**83.98**	**88.35**	**63.18**	**68.28**	**87.13**	**92.56**	**71.95**	**79.69**	**87.64**	**91.71**	
Gemma 2											
Temp 0.5	80.05	77.3	80.62	78.74	87.02	82.41	77.77	76.04	79.67	78.9	**79.85**
Temp 1	78.69	79.71	77.72	79.83	87.85	85.89	78.39	81.85	79.75	80.4	**81.01**
Temp 1.5	75.16	76.51	71.31	75.56	83.87	85.9	72.26	79.44	75.35	79.14	**77.45**
Mean	**77.97**	**77.84**	**76.55**	**78.04**	**86.25**	**84.73**	**76.14**	**79.11**	**78.25**	**79.48**	
Llama 3											
Temp 0.5	69.75	65.99	64.4	62.91	75.52	70.13	75.58	71.02	71.66	68.63	**69.56**
Temp 1	70.61	67.43	61.83	61.48	82.68	81.87	73.09	76.68	69.73	70.44	**71.59**
Temp 1.5	54.22	59.34	45.1	47.97	60.18	64.24	44.69	47.81	42.28	46.2	**51.2**
Mean	**64.86**	**64.25**	**57.11**	**57.45**	**72.8**	**72.08**	**64.45**	**65.17**	**61.22**	**61.76**	
Mixtral											
Temp 0.5	85.5	83.28	71.04	70.28	87.24	85.61	72.01	74.04	84.32	83.77	**79.71**
Temp 1	85.59	84.67	70.47	69.36	86.75	87.98	71.82	73.83	84.38	86.52	**80.14**
Temp 1.5	83.32	87.96	68.69	70.12	81.22	87.31	72.92	77.75	82.33	86.03	**79.77**
Mean	**84.81**	**85.31**	**70.07**	**69.92**	**85.07**	**86.97**	**72.25**	**75.2**	**83.68**	**85.44**	
Grand mean	**78.81**	**79.41**	**67.51**	**68.9**	**82.93**	**83.9**	**71.51**	**74.76**	**79**	**81.13**	

Bold in the table represent grand means

**Fig. 3:**
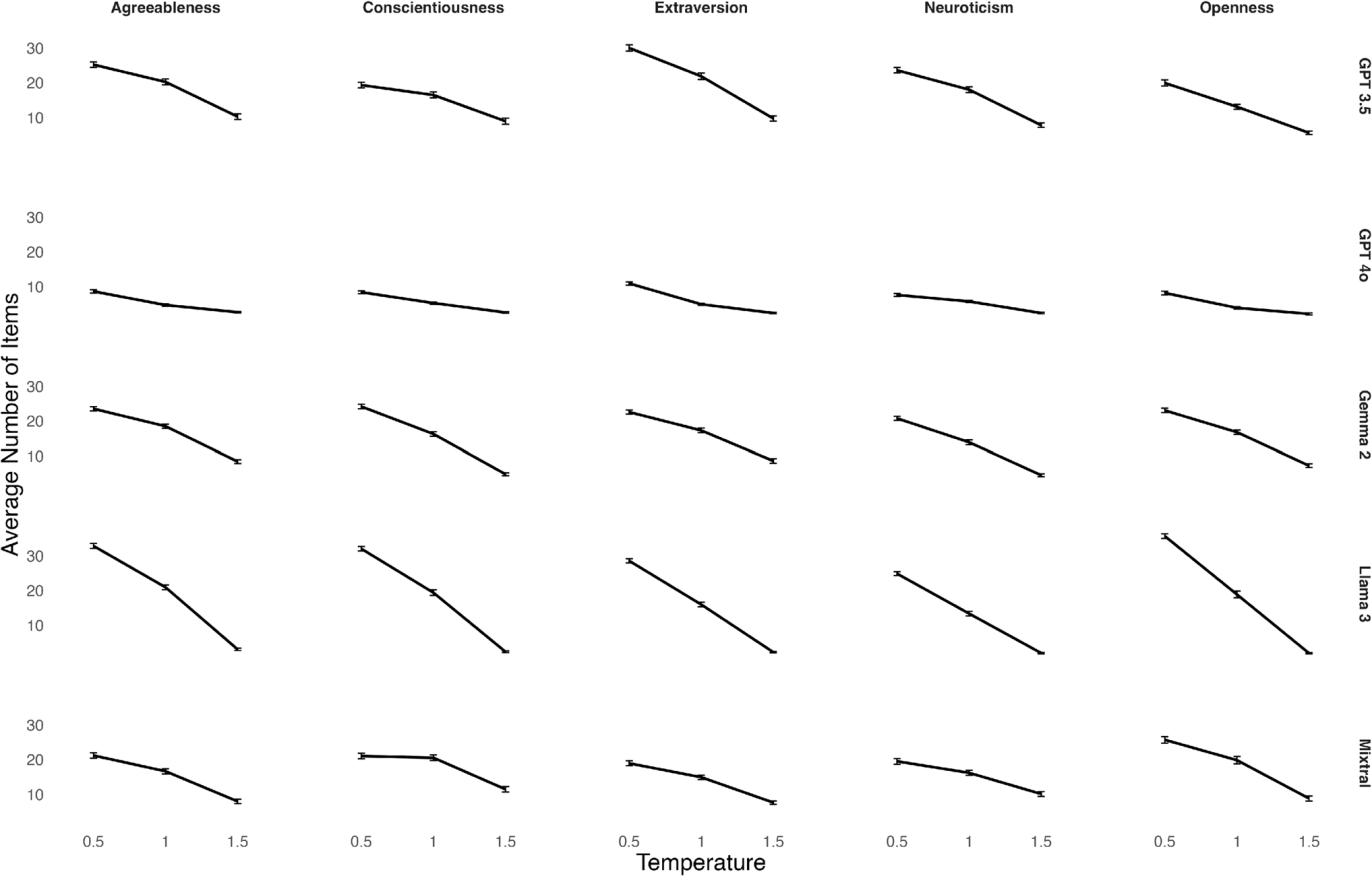
The relationship between the average number of redundant items removed in Step 3 of the pipeline and model temperature and item type

**Fig. 4:**
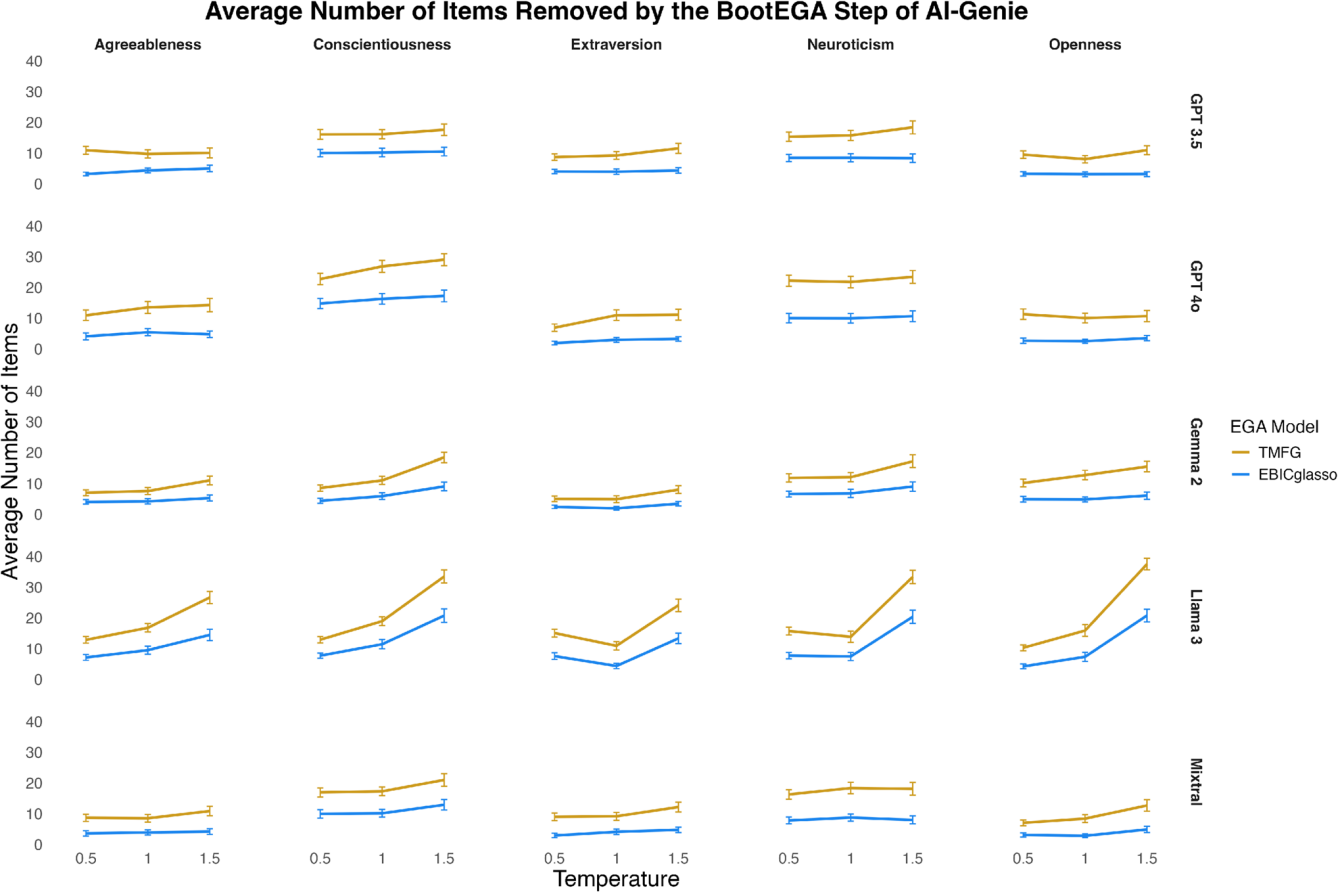
The relationship between the average number of items removed by the bootEGA step and model temperature, item type, and EGA model

For most models, baseline performance is comparable across all temperatures, as the difference between the highest and lowest NMI is fewer than 12 points. Llama 3, however, showed a more distinctive initial performance across temperatures. The lowest-temperature model was marginally worse than the others, with NMI values under 70, but the highest-temperature model performed much worse than any other model and temperature combination. Llama 3’s highest-temperature model had the worst average overall initial NMI of only 51.2, over 31.8 points lower than that of GPT-4o’s default-temperature model. Both of GPT-4o’s lower-temperature models showed high baseline performance with overall average NMIs above 83. Gemma 2’s default-temperature model also performed well with an NMI above 81. All other average overall NMIs hovered between 77 and 81.

### Step 3: Reduce redundancies

The next step in AI-GENIE is to remove redundant items using UVA (as described in Step 3 above) with a lowered cut-off value of 0.20. Because UVA operates on embedding vectors that encode contextual meaning, it can detect redundant item pairs that describe nearly identical behavior even when the items share no exact phrasing. For example, “I enjoy social events with lots of people” and “I thrive in large gatherings” could be flagged as redundant due to their semantic proximity in the embedding space, whereas a simple keyword matcher would miss such overlap.

This step of the pipeline is run iteratively until all items are unique. Typically, this process takes fewer than four rounds and may even be completed after a single round of UVA. For all experimental conditions, there appears to be an inverse relationship between model temperature and the average number of UVA sweeps (Fig. 10 in the Appendix). This is expected since lower-temperature models are more likely to be repetitive. This relationship is most extreme for Llama 3: an average of three or more rounds of UVA is required for its lowest-temperature model, while its highest-temperature model only needs about one and a half rounds (consistent with other high-temperature models).

As expected, a similar inverse relationship emerges when looking at the total number of redundant items removed across all UVA sweeps (Fig. 3). The relationship is most salient for Llama 3. Very few items were removed from GPT-4o across all temperatures. This suggests that the adaptive prompting approach (Lightman et al., 2023) to elicit unique items was most effective for this model, as most items were already unique prior to UVA, even at low temperatures.

### Step 4: Determine which embedding type to use

This step determines which embedding type (either sparse or full) to use for the remaining steps. For most experimental conditions, the full embeddings were used for a vast majority of samples (Fig. 11 in the Appendix). Notably, though, fewer than 54% of the samples used the full embeddings for the conscientious items generated by the lowest-temperature Llama 3 model. By contrast, over 91% of the samples targeting neuroticism that were generated using the highest-temperature GPT-4o model used the full embeddings.

### Step 5: Remove unstable items

Once the optimal embedding type is determined, bootEGA runs and repeats as necessary until all remaining items are stable (i.e., all item stabilities ≥ 0.75). As shown in Fig. 12 in the Appendix, the TMFG condition demands more rounds of bootEGA on average than EBICglasso. There appears to be a weak, direct relationship between model temperature and the number of bootEGA iterations, especially for conscientiousness items generated by Llama 3 when using TMFG, though this relationship is nearly absent for most conditions.

There does appear to be a stronger, direct relationship between the number of items removed in this step of the pipeline and model temperature for items generated by the Llama 3 model, especially when using TMFG (Fig. 4). Across all conditions, though, more items are discarded when using the TMFG algorithm, which is consistent with there being more rounds of bootEGA on average for this algorithm.

## Step 6: Review final item pool and note final NMI

The overall average final NMI for all models (except Llama 3) across all conditions exceeded 89 (Table 3). Gemma 2 and GPT-4o models yielded the highest overall average NMI of 94.46 and 93.05, respectively,^2^ though the margin was very slim (Llama 3 is once again the exception).

On the other hand, Llama 3’s lowest-temperature model had an overall average NMI of only 78.23, the default-temperature model performed almost at the level of its peers with an overall average NMI of 84.92, and the highest-temperature model showed the worst performance of only 71.24. Llama 3 still showed *improvement* in NMI after AI-GENIE item pool reduction. Regardless of model, our pipeline is equipped to handle artificially authored items.

**Table 3:** Mean NMI after AI-GENIE implementation

	AGR		CONSC		EXTR		NEUR		OPEN		Grand mean
	TMFG	GLASSO	TMFG	GLASSO	TMFG	GLASSO	TMFG	GLASSO	TMFG	GLASSO	
GPT-3.5											
Temp 0.5	93.58	91.01	87.31	87.36	93.61	93.12	88.82	86.94	94.84	93.45	**91**
Temp 1	94.46	92.3	89.47	85.53	95.91	95.1	90.24	87.38	95.92	94.36	**92.07**
Temp 1.5	93.81	90.75	86.31	81.35	96.26	94.58	88.36	87.8	94.03	91.84	**90.51**
Mean	**93.95**	**91.35**	**87.7**	**84.75**	**95.26**	**94.27**	**89.14**	**87.37**	**94.93**	**93.22**	
GPT-4o											
Temp 0.5	98.11	95.89	89.01	86.83	98.68	98.8	91.29	90.26	98.41	97.34	**94.46**
Temp 1	97.28	95.17	89.61	85.03	97.99	96.63	92.7	91.49	98.53	96.36	**94.08**
Temp 1.5	93.36	90.93	83.23	76.99	96.24	94.27	90.99	88.21	93.21	91.49	**89.89**
Mean	**96.25**	**94**	**87.28**	**82.95**	**97.63**	**96.57**	**91.66**	**89.99**	**96.71**	**95.06**	
Gemma 2											
Temp 0.5	90.68	88.84	92.46	90.75	97.99	97.1	92.68	93.34	94.39	92.28	**93.05**
Temp 1	88.52	89.73	92.41	91.07	97.44	96.35	93.08	93.16	95.28	91.42	**92.85**
Temp 1.5	86.3	84.06	89.65	84.77	94.98	94.27	90.1	88.57	91.2	88.02	**89.19**
Mean	**88.5**	**87.54**	**91.51**	**88.87**	**96.8**	**95.91**	**91.96**	**91.69**	**93.62**	**90.57**	
Llama 3											
Temp 0.5	79.48	79.4	68.33	66.94	84.73	80.66	83.4	85.14	80.13	74.14	**78.23**
Temp 1	87.96	82.19	76.79	72.31	94.69	92.09	89.16	88.03	83.83	82.2	**84.92**
Temp 1.5	78.96	75.32	64.86	60.6	81.73	77.56	72.81	65.83	72.06	62.64	**71.24**
Mean	**82.13**	**78.97**	**69.99**	**66.62**	**87.05**	**83.44**	**81.79**	**79.66**	**78.67**	**73**	
Mixtral											
Temp 0.5	94.87	93.33	88.56	81.62	96.62	96.25	84.98	83.9	95.28	92.44	**90.78**
Temp 1	95.9	93.79	89.15	81.24	96.72	96.94	85.87	85.52	96.62	93.92	**91.57**
Temp 1.5	96.35	93.79	86.18	84.58	95.07	93.9	90.98	89.31	95.57	93.5	**91.92**
Mean	**95.71**	**93.64**	**87.96**	**82.48**	**96.14**	**95.7**	**87.28**	**86.24**	**95.82**	**93.28**	
Grand mean	**91.31**	**89.1**	**84.89**	**81.13**	**94.58**	**93.18**	**88.36**	**86.99**	**91.95**	**89.03**	

Bold in the table represent grand means

**Fig. 5:**
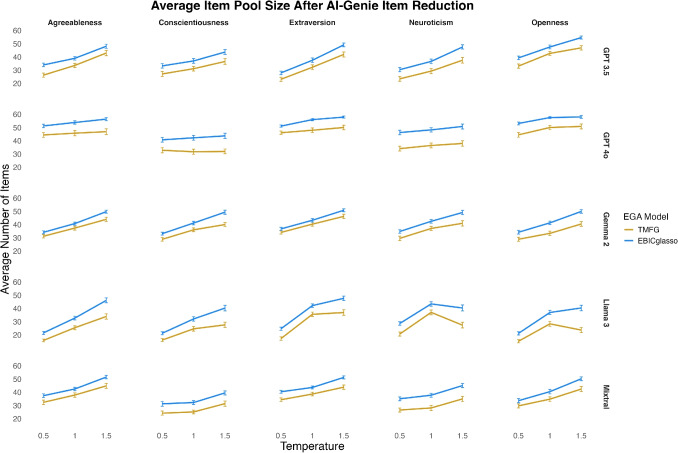
The relationship between the average item pool size after AI-Genie reduction and model temperature, item type, and EGA model

Average final item pool sizes range from roughly 15 to 58 items, depending on the condition. For most models, there is a direct relationship between model temperature and average final item pool size (Fig. 5). For extraversion, neuroticism, and openness items generated by Llama 3, the highest retention rate occurs at the default-temperature value rather than a temperature of 1.5, which is especially apparent in the TMFG condition. Notably, TMFG retains fewer items than EBICglasso, regardless of condition. However, while still present, this difference is largely negligible for items generated by Gemma 2, especially for the trait extraversion.

AI-GENIE consistently improved NMI scores across all models and temperature settings, with the TMFG algorithm tending to build more refined final item pools (fewer items, comparable or higher NMI) relative to EBICglasso.

## Example of practical use

To illustrate a practical application of AI-GENIE, we present an example in which a researcher wishes to create a new Big Five personality scale mimicking John and Srivastava’s (1999) assessment. Note that while this example uses LLM-generated items, AI-GENIE’s pipeline can equally refine human-authored item pools.

### Generating items

GPT-4o (default temperature and top p) was used to generate 64 items per Big Five trait (320 total). The prompt employed the same techniques described in the simulation: role prompting (expert psychometrician persona), few-shot prompting (ten example items from John and Srivastava’s (1999) scale, with the instruction to emulate quality, not content), attribute-specific generation (two items per attribute), and explicit formatting constraints. The full prompt text is available in the Appendix; for detailed guidance on prompt engineering within AI-GENIE, see Russell-Lasalandra and Golino (2026).

### Using AI-Genie item pool reduction

**Fig. 6:**
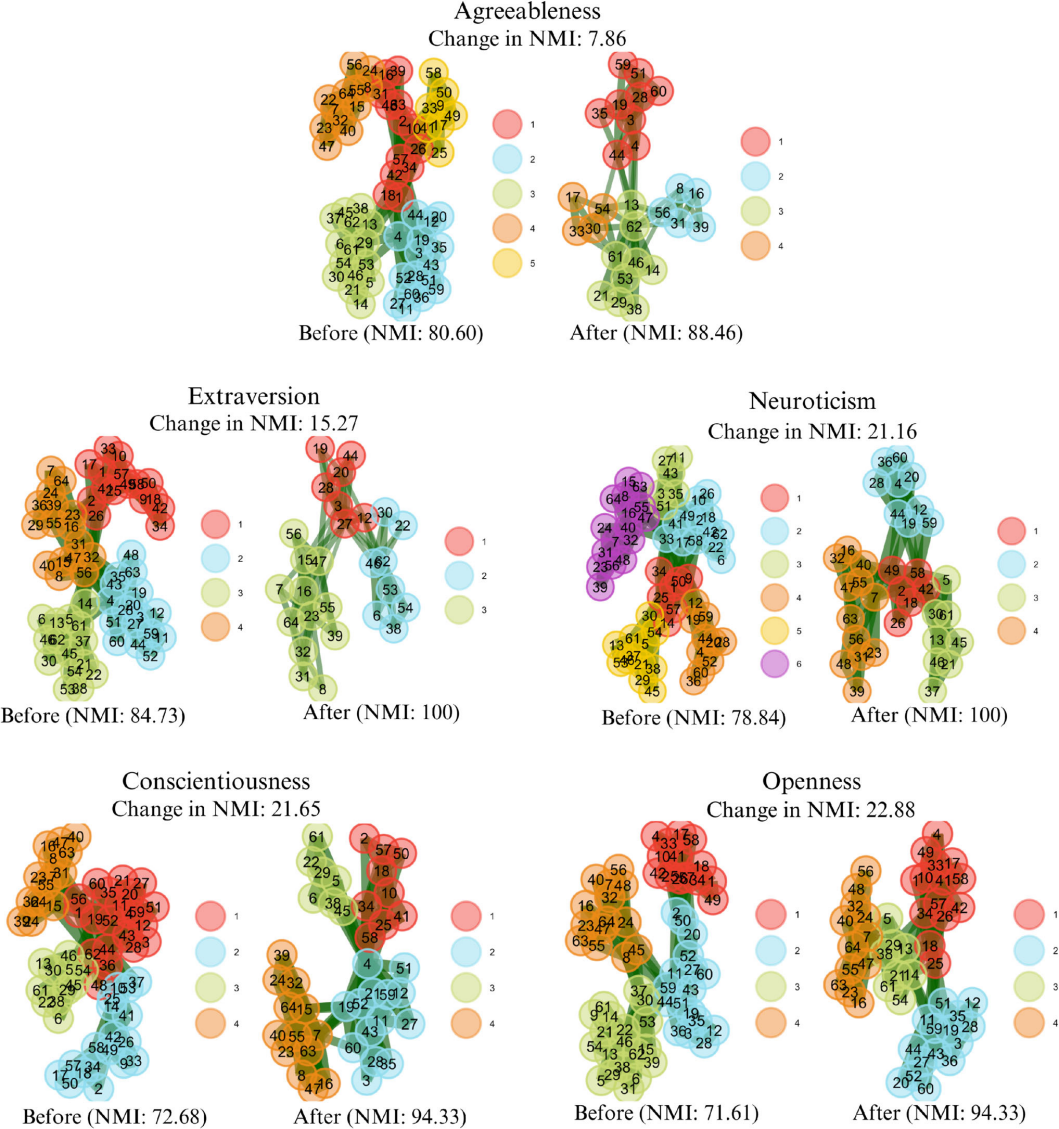
The network plots before vs. after AI-Genie implementation for all Big Five personality traits for the empirical example generated by GPT-4o

**Fig. 7:**
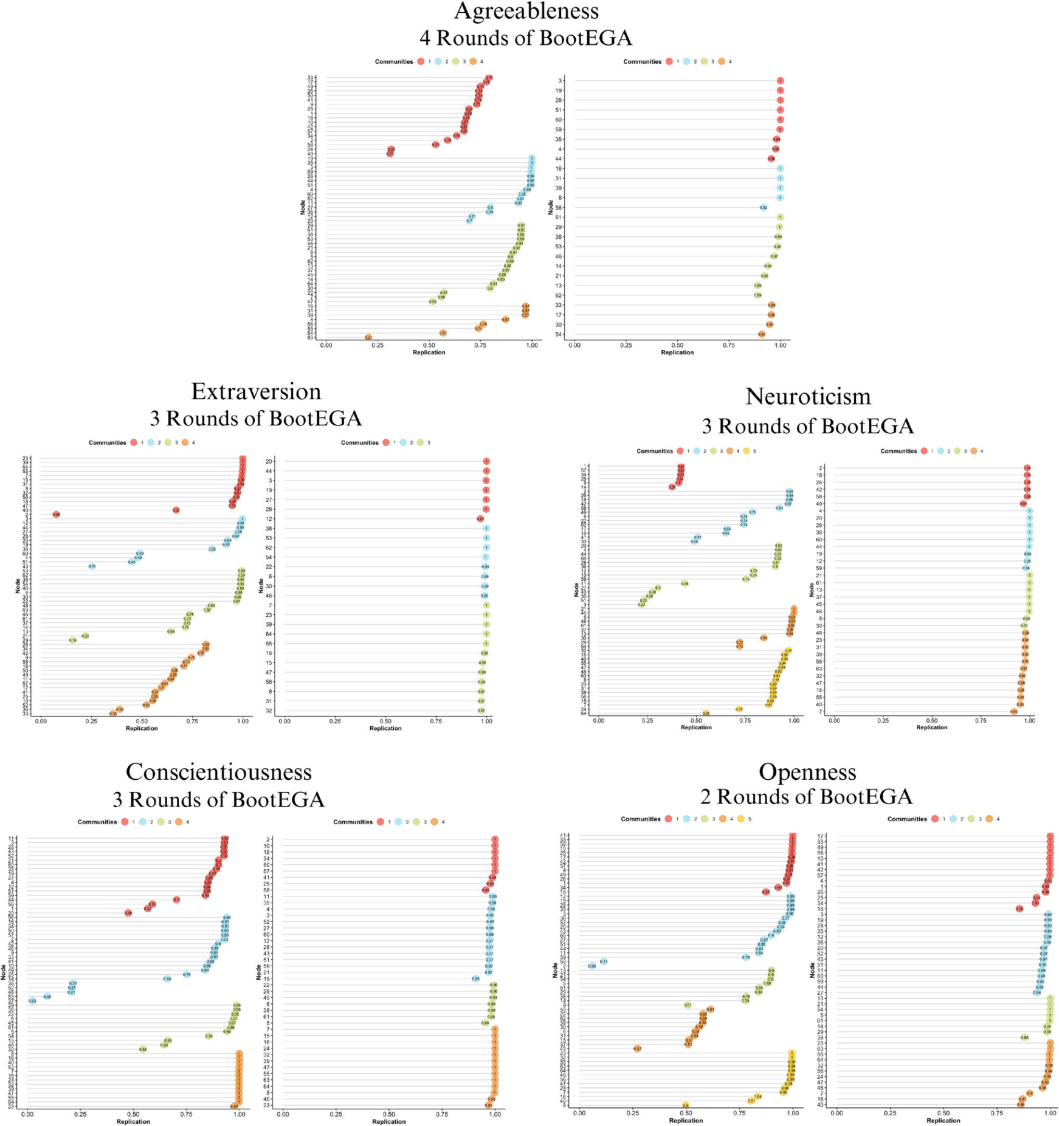
The stability plots before vs. after AI-Genie implementation for all Big Five personality traits for the empirical example generated by GPT-4o

For this example, only TMFG was used with EGA to build the networks. The item pool shrunk from 320 items to 181 items: 27 related to agreeableness, 43 related to conscientiousness, 27 related to extraversion, 35 related to neuroticism, and 49 related to openness. The NMI improved for all traits after pipeline reduction (Fig. 6). The improvement can be further illustrated by the item stability plots, all of which show that the communities become highly stable after several rounds of bootEGA (Fig. 7). The largest gains were for items related to neuroticism and extraversion, where the NMI after reduction reached 100 (perfect community detection). The smallest gain was for items related to agreeableness with a final NMI of 88.46, though this still represents an improvement of over seven NMI points.

## Empirical validation

To demonstrate the validity of the surveys produced by AI-GENIE, we created five new Big Five personality surveys using Gemma 2, GPT-3.5, GPT-4o, Llama 3, and Mixtral. The same prompt and instructions were used for each model (the full prompt is provided in the Appendix). Each model generated at least 40 items using a temperature of 1 and the EBICglasso method was used to validate the items in AI-GENIE. The resulting surveys had items ranging from 28 (Gemma 2) to 35 (GPT-4o) with at least four items per Big Five trait.

### Participants

Five nationally representative samples in the United States were recruited from Prolific. Each sample consisted of approximately 1000 people (final Ns ranged from 989 to 997 after exclusions) who completed one of the five Big Five surveys created and validated by AI-GENIE. Participants were compensated at $12/hour, with the median duration to completion for each sample ranging from 3-4 minutes. Participants were excluded from analysis if they demonstrated straight-line responding (zero variance in their responses) or had responded with a single response (e.g., “Agree”) for 95% or more of the items. All surveys were administered independently over Qualtrics and, based on exclusion criteria on Prolific, people were only allowed to complete one of the five surveys. Participants provided consent, completed a survey, and concluded with a set of demographics questions. Full descriptive statistics for each sample are provided in Table 5 of the Appendix. This study and subsequent surveys were all approved by the [masked] University’s institutional review board.

### Materials

All surveys were completed using a five-point Likert scale ranging from ‘1’ (Strongly Disagree) to ‘5’ (Strongly Agree) with a midpoint of ‘3’ (Neither Disagree nor Agree). People were provided with a response option of ‘Not Applicable’ in case any of the items did not apply to them. All survey items are provided on our OSF page.

### Analyses

To provide an evaluation of structural validation, we performed two procedures: UVA and bootstrap EGA. One goal of AI-GENIE is to reduce redundancies. UVA assessed the extent to which there were moderate (≥0.25) and large (≥0.30) redundancies. For the robustness of the structure, bootEGA provided item stability estimates with the goal of achieving ≥0.75 item stability. Finally, to evaluate the extent to which the empirical results aligned with the embedding item placements, we computed NMI between the embedding structure and the empirically derived structure. To provide traditional psychometric benchmarks, we also conducted confirmatory factor analyses (CFA) using weighted least-squares mean and variance adjusted estimation (WLSMV; Muthén et al., 1997) and computed McDonald’s (ω) as a measure of internal consistency for each factor (see Table 6 in the Appendix). These values were comparable or better than what is typically observed for personality inventories under CFA (Hopwood & Donnellan, 2010).

## Results

**Fig. 8:**
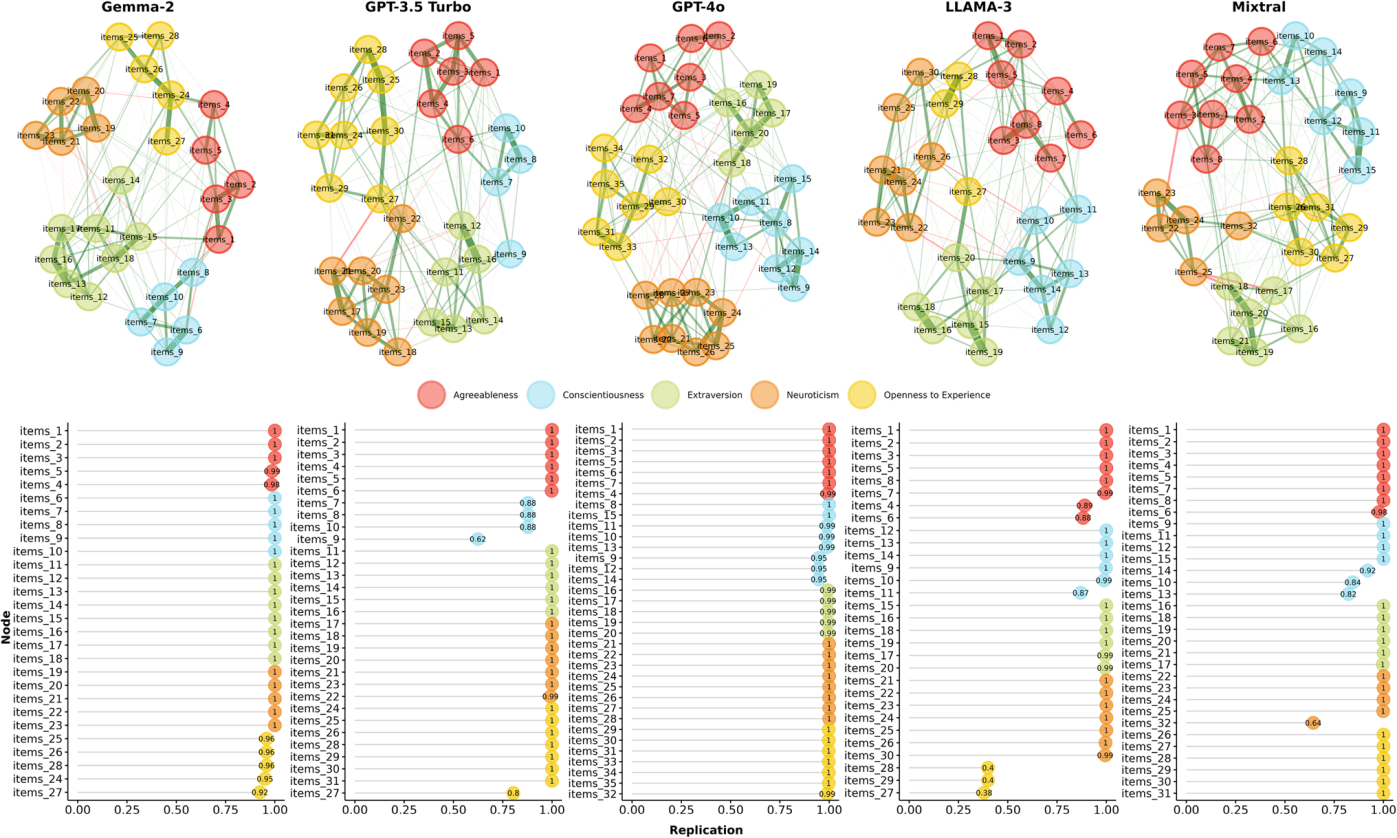
Exploratory graph analysis (*top row*) and item stability results (bottom row) for each LLM used in the study (across the columns). *Green* and *red lines* indicate positive and negative partial correlations, respectively. The *thickness of the lines* indicates size of partial correlation. The *color of the nodes* represents the community (dimension) each item belongs to

The empirical results are presented in Table 4 and Fig. 8. The number of items varied from 28–35 in the post-processing of AI-GENIE. The number of moderate and large redundancies varied across the LLMs, with all having at least two moderate redundancies. The two models that had the fewest moderate redundancies, GPT-4o and Llama 3, also had the most large redundancies, with 2 and 3, respectively. In terms of sum total redundancies, Gemma 2 led all models with the fewest (3) followed by GPT-4o (4) and Mixtral (4).

**Table 4:** UVA = unique variable analysis with moderate (0.25) and large (0.30) redundancies (lower counts are better); NMI = normalized mutual information (larger values are better)

		UVA		NMI	
LLM	Items	≥0.25	≥0.30	Theoretical	AI-GENIE
Gemma 2	28	3	0	1.00	0.94
GPT-3.5	31	4	1	1.00	1.00
GPT-4o	35	2	2	1.00	1.00
Llama 3	30	2	3	0.92	0.94
Mixtral	32	3	1	0.95	0.89

The structures identified by EGA (top row of Fig. 8) all show five distinct communities corresponding to the Big Five dimensions: agreeableness, conscientiousness, extraversion, neuroticism, and openness to experience. There was a perfect match for three of the five models: Gemma 2, GPT-3.5, and GPT-4o (Table 4). Mixtral (NMI = 95) and Llama 3 (NMI = 92) had one (items_32) and two (items_26 and items_30) items that were placed differently than the theoretical item placements. Only GPT-3.5 and GPT-4o were perfectly placed with AI-GENIE’s placements, followed by Gemma 2 and Llama 3 (both NMIs = 94) and Mixtral (NMI = 89). A source of difference is likely to lie in the embeddings’ meanings and the participants’ interpretations of the items. These results show congruence with the theoretical and AI-GENIE item placements.

The robustness of these structures was evaluated by Bootstrap EGA using resampling with replacement. The item stabilities are reported in the bottom row of Fig. 8. Both Gemma 2 and GPT-4o had high item stabilities, with items replicating in the original EGA’s dimension at least 90% of the time. Both GPT-3.5 and Mixtral had only one problematic item (<0.75). Llama 3 had several items with low stability (items_27, items_28, items_29), which are all openness to experience items. A key part of their low stability is likely that the other two openness to experience items (items_26 and items_30) were pulled into neuroticism.

Gemma 2 and GPT-4o performed best in terms of minimizing redundancies, maximizing similarity to the embedding structure, and maximizing the item stabilities within those dimensions. GPT-3.5 and Mixtral also performed well, with only one problematic item each with respect to item stabilities. Llama 3, consistent with the simulations, was the worst of the models tested in terms of all three facets (redundancy, embedding similarity, and structural integrity). CFA model fit indices and McDonald’s ω internal consistency estimates for each LLM-generated scale are reported in Table 6 of the Appendix. The AI-GENIE-generated scales showed fit comparable to the BFI under the same modeling conditions, consistent with what is typically observed for personality inventories under CFA (Hopwood & Donnellan, 2010).

**Fig. 9:**
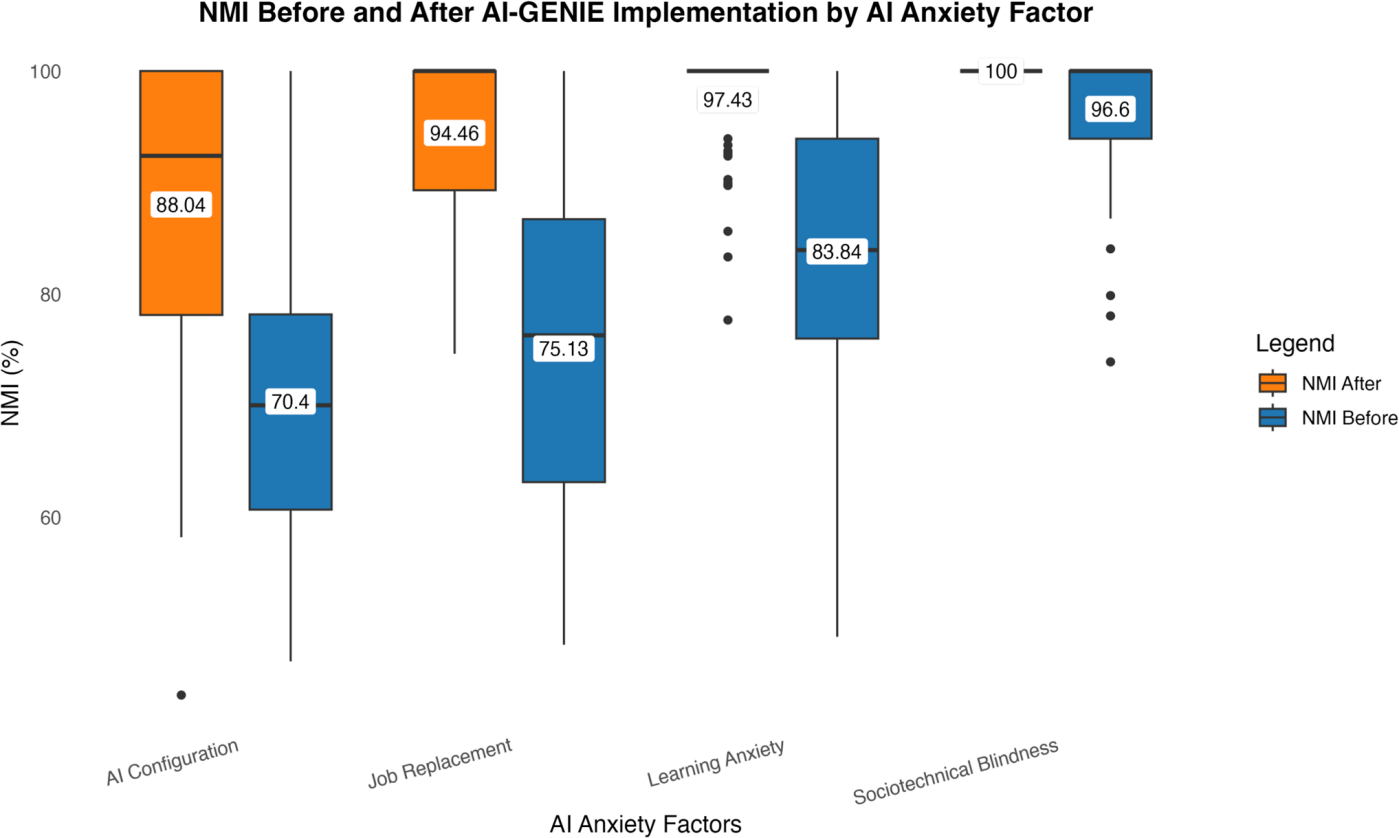
The distribution of NMI scores before and after AI-GENIE item pool reduction on the emerging construct AI anxiety. *Note*: the value displayed on the *box and whisker plot* reflects the approximate value and placement of the distribution MEAN, rather than the median

## AI-GENIE on an emerging construct: A small demonstration

An important limitation of the current demonstration is its reliance on the Big Five personality model, a well-established construct with extensive representation in the psychological literature and, likely, in the training data of LLMs. While this choice was deliberate (since it provides a validated benchmark against which to evaluate AI-GENIE’s performance), it does not demonstrate the method’s utility for novel or emerging constructs where item generation assistance would be most valuable.

As such, we have also included a small-scale supplemental simulation applying AI-GENIE to the emerging psychological construct of AI anxiety (AIA). This emerging construct encompasses the multifaceted anxieties we experience as AI rapidly transforms how we work, learn, and generally function in daily life (Wang, 2022; Yang, 2025). No scale that specifically addresses feelings of uncertainty and hopelessness in the face of AI had been developed until very recently (Yang, 2025). Current studies are still adapting and validating the scale across different populations ((Liu, 2025) looks at AIA in Chinese teachers, while Güven et al. (2024) examines AIA in medical students). Thus, while some AIA scales exist, this construct lacks comprehensive validation across diverse contexts (Li, 2020) and is much less established than the Big Five.

For our small-scale simulation, we generated items based on the four-factor structure identified by Wang and Wang (2022): learning anxiety, job replacement, sociotechnical blindness, and AI configuration. The definitions of each of these factors were provided to the LLM of choice, GPT-4o:

1. *Learning anxiety*^3^ can be described as cognitive overwhelm with AI complexity, which includes perceiving one’s knowledge as insufficient for AI demands and feeling daunted by the pace of AI advancement.
2. *Job replacement* can be described as fear of professional obsolescence, which includes fearing AI will eliminate one’s professional role, believing one’s skills can be automated, and experiencing uncertainty about career stability.
3. *Sociotechnical blindness* can be described as concern about societal AI impacts, which includes loss of autonomy to AI systems, concerns about AI-enabled privacy violations, and worrying about over-reliance on AI technology.
4. *AI configuration* can be described as anxiety about AI system opacity, which includes lacking confidence in AI decision-making, confusion about how AI systems operate, and doubting AI’s reliability and safety.

Based on these factor definitions, the following attributes were identified:

1. *Learning anxiety*: overwhelmed, inadequacy, intimidated
2. *Job replacement*: threatened, replaceable, insecure
3. *Sociotechnical blindness*: powerless, overly dependent, surveilled
4. *AI configuration*: distrustful, uncertain, vulnerable

For this small-scale simulation, 50 samples of at least 60 items were generated for each of the four factors using GPT-4o (at the default temperature). For each sample, both TMFG and EBICglasso were tested, and the EGA algorithm that could better identify the factors’ attributes was retained.

As can be seen in Fig. 9, the NMI post-item pool reduction is not only better than the NMI pre-reduction on average for each of the four factors, but also much more consistent. The post-reduction mean NMI reached 100 for the sociotechnical blindness condition. Even the worst-performing factor had a post-reduction average NMI of 88, an improvement over the 70 figure pre-reduction. These results suggest that AI-GENIE performed as expected, at least in simulation, when tasked with drafting items for a less-established construct.

## Discussion

Our study presents the AI-GENIE approach to select, reduce, and structurally validate an artificially-authored item pool in the context of the Big Five personality traits. The in-silico structural validity of the items closely matched the results obtained in nationally representative samples of US adults for the best-performing LLM models. In six steps, our approach combines LLMs, genAI, and network psychometrics to augment the labor-intensive process of scale development. The results show consistent improvement in NMI scores across all models and temperature settings, suggesting that the approach can improve the coherence and precision of item pools regardless of model choice. Situated within the broader program of Generative psychometrics, AI-GENIE contributes a single integrated pipeline for item generation, selection, and structural validation, complementing parallel work on embedding-based scale abbreviation, nomological mapping, validity diagnostics, and synthetic respondent modeling.

This work is relevant to the growing demand for psychological assessments across diverse contexts, including clinical, educational, and organizational settings. Traditional scale development can take years and cost thousands of dollars, requiring multiple experts (Fenn et al., 2020; Kyriazos & Stalikas, 2018). AI-GENIE lowers these barriers by using LLM embeddings to represent item meaning in a high-dimensional space and network psychometric methods (UVA, EGA, bootEGA) to automate redundancy detection, dimensionality assessment, and stability-based item selection. Unlike traditional approaches that rely on the subjective judgment of experts for item selection, AI-GENIE uses stability metrics and community detection algorithms to inform retention decisions, allowing experienced methodologists to focus their expertise on higher-level decisions (e.g., connecting content and structure to measurement and theory).

### Limitations and future directions

#### Implications of open-source models

Perhaps the most notable finding from our extended analyses using DeepSeek and GPT OSS 120b and 20b is that GPT OSS 120b matched or exceeded the performance of GPT-4o, achieving average final NMIs of 94.57-95.35 across temperatures (see Table 11 in the Appendix). This finding suggests that open-source models may now rival the psychometric utility of proprietary systems in the AI-GENIE framework. This finding has both practical and ethical significance: open-source availability allows researchers to download and run models locally, enabling item generation workflows to remain entirely on secure, institutional hardware rather than relying on external servers. While these results should be interpreted cautiously, as we have not yet conducted empirical validations for GPT OSS 120b, they highlight a promising avenue for democratizing psychological scale development with high-performing, openly available LLMs.

#### AI-GENIE in less established contexts

Beyond our own simulations, AI-GENIE has now been applied independently to develop and validate a published psychological scale in a novel domain. Kopka et al. (2025) used AI-GENIE to construct the Trust in AI-Generated Health Advice scale (TAIGHA), validating it with 385 UK participants in a symptom-assessment scenario. Their final ten-item scale showed excellent confirmatory factor analytic fit (CFI = 0.98, RMSEA = 0.07, SRMR = 0.03), high internal consistency (α = 0.95), and strong convergent and criterion validity, including a stronger correlation with reliance on AI advice than the established Trust in Automated Systems Survey. Their workflow combined AI-GENIE’s automated item generation and structural filtering with subsequent expert content validation and lay face validation. The authors explicitly recommend this hybrid procedure: AI-GENIE for efficient item generation and structural validation, followed by traditional content and face validity steps to ensure theoretical alignment. Their successful application to a novel construct in a domain quite different from personality measurement provides external evidence that AI-GENIE generalizes beyond the Big Five. This research reinforces AI-GENIE as a tool that accelerates specific phases of scale development.

Although promising, there are several avenues to evaluate and improve AI-GENIE. Generating items for less-established constructs may present additional challenges, as LLMs may have limited or even nonexistent training data about emerging psychological phenomena, potentially resulting in less diverse or less theoretically grounded item pools. Future research should systematically evaluate AI-GENIE’s performance across constructs varying in their theoretical maturity and representation in training corpora. However, we do demonstrate in our small-scale simulation that AI-GENIE shows promise for the emerging construct of AI anxiety. Even so, further research is needed to assess AI-GENIE’s capabilities and limitations with emerging constructs, as we lack the same empirical evidence available for the personality simulation.

#### Questions of validity and human intervention

As noted in the Introduction, AI-GENIE addresses the “structural phase” of validation (Christensen et al., 2020), i.e., evidence based on internal structure (Aera et al., 1999; Rios & Wells, 2014), and does not provide evidence for the remaining four sources (test content, response processes, relations to other variables, or consequences of testing). Content validity still requires expert evaluation (Haynes et al., 1995), response process evidence requires cognitive interviews (Padilla Garcia et al., 2014), and convergent, discriminant, and criterion-related validity demand additional data collection (Campbell & Fiske, 1959). AI-GENIE should therefore be positioned as a tool that accelerates specific phases within established scale development frameworks (Devellis and Thorpe 2021; e.g., Hinkin, 1998; Lambert and Newman, 2023), not as a comprehensive validation system, consistent with calls for improved measurement practices (Flake et al., 2022; Fried et al., 2022)).

Recent work by Cummins et al. (2026) illustrates the importance of going beyond face validity when evaluating LLM-generated items. In two studies of multiple-choice exam questions for university courses, lecturers rated LLM-generated items as broadly comparable in quality to human-authored items, yet IRT analysis revealed that the same items were systematically easier and less discriminating than human-authored counterparts. Their findings suggest item quality and psychometric performance can diverge sharply when LLM-based item generation is not paired with rigorous structural and parametric evaluation. AI-GENIE addresses precisely this gap by providing structural-based evidence for item quality, so scale developers need not rely on face-validity judgments alone. In our own empirical validation, we similarly moved beyond face-validity judgments by administering AI-GENIE-generated Big Five scales to five samples (*N* = 4964) and comparing the recovered dimensional structure against the embedding-derived Big Five. Three of the five models achieved full embedding alignment (NMI = 100), and the remaining two reached 92 and 95, with item stabilities exceeding 0.75 for most models and few redundancies. These results suggest that the structural validation phase of AI-GENIE manifests in real respondent data, at least for well-established constructs. We note, however, that AI-GENIE’s structural validation does not substitute for IRT-based examination of item difficulty and discrimination, which remains an important complementary step. Future extensions of AI-GENIE could incorporate post-selection IRT analysis (e.g., 2-PL models for difficulty and discrimination, as in Cummins et al. (2026)) as a parametric companion to structural validation.

Importantly, while we demonstrate AI-GENIE using LLM-generated items, the core methodology is agnostic to item source and can be equally applied to human-authored item pools. Whether applied to AI-generated or human-authored items, AI-GENIE is designed to augment, not replace, the careful scientific judgment that underpins rigorous scale development (Borsboom, 2005).

#### Applicability of network psychometric methods to embedding data

A reasonable concern is whether EGA, UVA, and bootEGA, which were originally developed and validated on person-level response data, are appropriate for the embedding matrices used in AI-GENIE. In our pipeline, the data matrix is transposed relative to traditional applications. Rows represent embedding dimensions and columns represent items, rather than respondents by items. The mathematical operations underlying these methods (network estimation, community detection, and topological overlap) do not inherently require that observations be persons. Rather, they require a matrix of continuous variables from which a meaningful correlation or association structure can be derived. Embedding matrices satisfy this requirement, as semantically related items produce correlated patterns across embedding dimensions. Recent simulation evidence addresses this question. Garrido et al. (2025) compared EGA and PCA on embedding-based similarity matrices and found that PCA severely overestimates dimensionality in this context, while EGA-based approaches achieve near-perfect dimensional recovery. These findings provide direct empirical support for the use of network psychometric methods on embedding data as implemented in AI-GENIE. Nevertheless, further methodological work examining the statistical properties of embedding matrices and their implications for network estimation is warranted.

#### Limitations of our empirical validation

The current empirical validation is limited to U.S. samples and English-language item generation. This is particularly important given psychology’s ongoing challenges with overreliance on WEIRD (Western, Educated, Industrialized, Rich, and Democratic) populations (Henrich et al., 2010). We strongly caution against assuming that AI-GENIE’s performance would generalize to other populations, languages, or cultural contexts without empirical validation. Language models are trained predominantly on English-language text from Western sources, potentially embedding cultural biases that could affect item generation for non-Western constructs or populations (Bender et al., 2021). Future research can prioritize evaluating AI-GENIE performance across non-WEIRD populations and non-English languages.

#### Potential areas for methodological improvement

The parameters of both the genAI (e.g., temperature) and network psychometric approaches (e.g., wTO) could benefit from further evaluation. The chosen wTO cut-off value used in this study (0.20) was largely determined through trial and error. Both the temperature parameter of genAI and the wTO cut-off value can have significant consequences on the final item pool. Rather than a direct limitation, the temperature and wTO values could be leveraged to produce scales with varying breadth (temperature) and different lengths (wTO). Lower temperatures could lead to item pools with a narrower focus on a measurement target, whereas higher temperatures could lead to item pools that are broad and diverse. Lower wTO values could lead to smaller item pools by eliminating more variables as redundant, whereas higher wTO values could lead to larger item pools by allowing more redundant items to remain. These two parameters in combination could be used to generate item pools that match a researcher’s desired breadth and length of their scale. Future work is necessary to determine the extent to which these two parameters are tunable to meet this aim.

Additionally, we should address the limitations of community detection, a crucial step of our process. Community detection is a central but inherently ill-posed problem in psychometric network analysis, where different algorithms yield divergent clusterings due to differing assumptions about what constitutes a “community” (Fortunato, 2016). For our simulation, we selected the Walktrap algorithm due to its strong empirical performance in previous simulation studies using psychological data generated from factor models, which is a context closely aligned with our own (Christensen et al., 2024; Golino et al., 2020). That said, we acknowledge the broader variability across community detection methods. Future work should more systematically evaluate the sensitivity of AI-GENIE’s outputs to different community detection strategies.

#### Importance of prompting

The quality of the prompt used to generate items has a direct impact on item pool quality. Less precise prompts could result in lower-quality item pools, potentially impacting the pipeline’s efficacy. In practice, researchers should elaborate thoroughly on the measurement target, specifying item characteristics (e.g., grammatical structure, reading level), providing theoretical definitions, and positioning the construct within its nomological network (Cronbach, 1955). A detailed examination of how prompt engineering strategies shape item quality within the AI-GENIE framework is provided in Russell-Lasalandra and Golino (2026).

#### Addressing concerns of accidental plagiarism

There are two important considerations that address concerns about the originality of AI-generated items. First, LLMs do not copy text verbatim from their training data; instead, they operate through learned patterns and probabilistic generation, similar to how humans form novel sentences (Bender et al., 2021; Brown et al., 2020). This generative process inherently produces novel combinations of words and ideas rather than reproducing existing items wholesale. Researchers using AI-GENIE can verify the semantic uniqueness of generated items using tools such as the Semantic Scale Network (Rosenbusch et al., 2020). This online application uses latent semantic analysis to detect overlap between scales, allowing researchers to ensure that generated items are semantically distinct from existing scales. Additionally, researchers can cross-reference generated items against public databases like the International Personality Item Pool (Goldberg et al., 2006) and proprietary databases such as PsycTESTS. Researchers can and should implement verification procedures to ensure the originality and value of AI-generated psychological items.

#### Final thoughts

This study introduced AI-GENIE, a method to automate item generation, selection, and structural validation in psychometric scale development. The AI-GENIE methodology has been integrated into an R package called AIGENIE available on the R Universe (see Russell-Lasalandra et al. (2026) for detailed instructions and guidance). By combining genAI, LLM embeddings, and network psychometrics, the approach aims to reduce the time and expertise required for early-stage scale development. The results show that AI-GENIE can refine AI-generated item pools, producing non-redundant items that align with intended dimensional structures. Model temperature influenced the diversity of AI-generated items: higher temperatures generally led to more varied responses, though with greater variability in initial NMI scores for certain models. Among the models tested, GPT-3.5 retained the most stable items and achieved the highest final NMI scores, suggesting that even older LLM versions can perform well in this context.

To validate these simulation findings with real-world data, we administered five AI-GENIE-generated Big Five surveys to nationally representative U.S. samples totaling 4964 participants. The empirical results showed that three of the five models (Gemma 2, GPT-3.5, and GPT-4o) achieved full embedding alignment (NMI = 100), while Mixtral and Llama 3 showed high alignment (NMIs of 95 and 92, respectively). Item stabilities exceeded 0.75 for most models, with few redundancies. These empirical findings suggest that AI-GENIE can produce structurally coherent instruments from AI-generated items.

By reducing the time, cost, and human resources required for early-stage scale development, AI-GENIE may make the process more accessible to researchers across various domains. The ability to generate and structurally evaluate large item pools in silico could broaden the availability of psychometric assessments, particularly for constructs where expert resources are limited. The methodology’s performance on the Big Five personality traits suggests that it may be applicable to other psychological constructs, though this remains to be tested empirically. More broadly, we hope AI-GENIE will serve as one building block within the emerging program of Generative psychometrics, alongside complementary work on prompt engineering for item authoring (Russell-Lasalandra, 2026), dimensionality recovery from embedding spaces (Garrido & Russell-Lasalandra, 2025), and the wider use of LLMs and text embeddings as scalable tools for psychological measurement.

## Figures and Tables

**Fig. 10:**
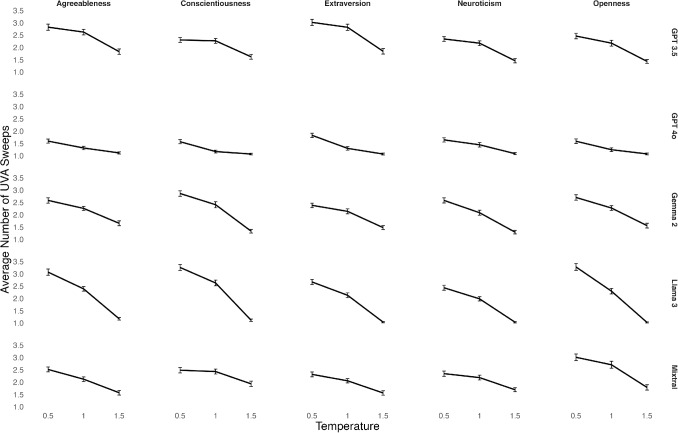
The relationship between the average number of UVA sweeps and model temperature and item type

**Fig. 11:**
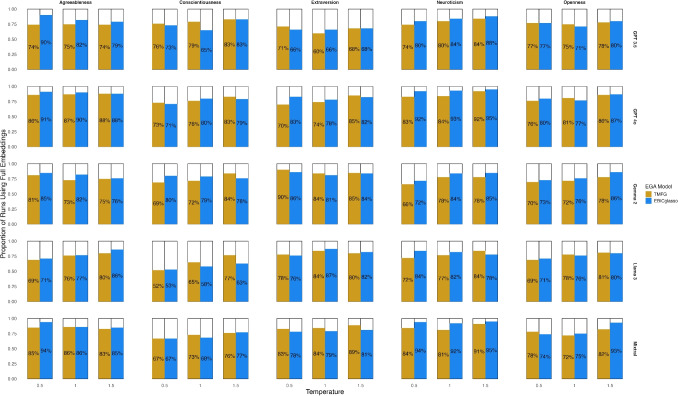
The relationship between the proportion of runs where the full embeddings were used and model temperature, item type, and EGA model

**Fig. 12:**
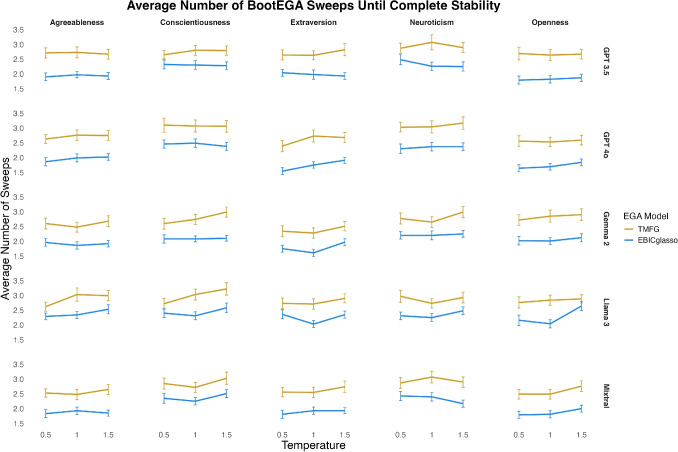
The relationship between the average rounds of bootEGA and model temperature, item type, and EGA model

**Fig. 13:**
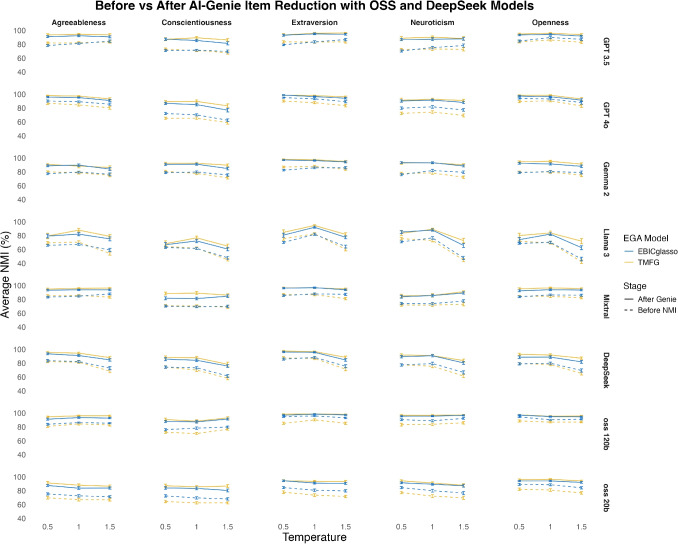
The relationship between the initial NMI (*dashed*), final NMI (*solid*), model temperature, LLM model, EGA model, and item type. This plot also includes the DeepSeek and OSS models, which were added to the initial simulation

**Fig. 14:**
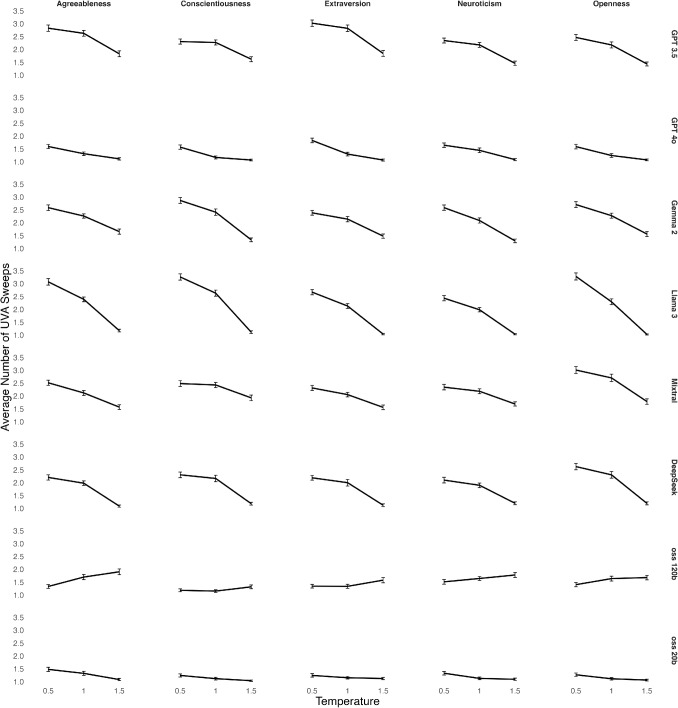
The relationship between the average number of UVA sweeps and model temperature and item type. This plot includes the DeepSeek and OSS models

**Fig. 15:**
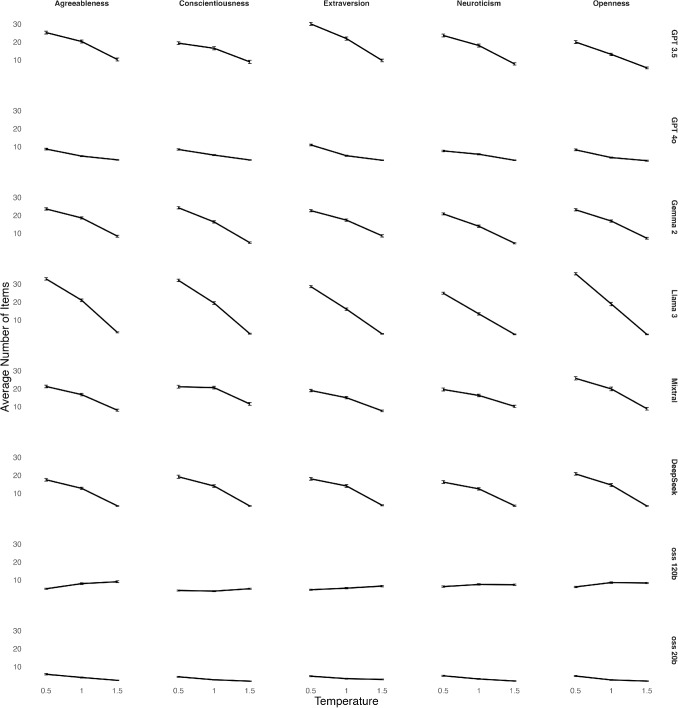
The relationship between the average number of redundant items removed in Step 3 of the pipeline and model temperature and item type. This plot includes the DeepSeek and OSS models

**Fig. 16:**
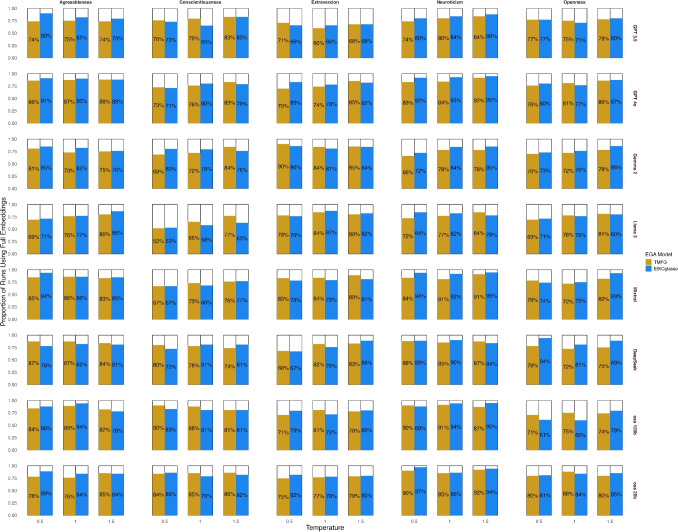
The relationship between the proportion of runs where the full embeddings were used and model temperature, item type, and EGA model. This plot includes the DeepSeek and OSS models

**Fig. 17:**
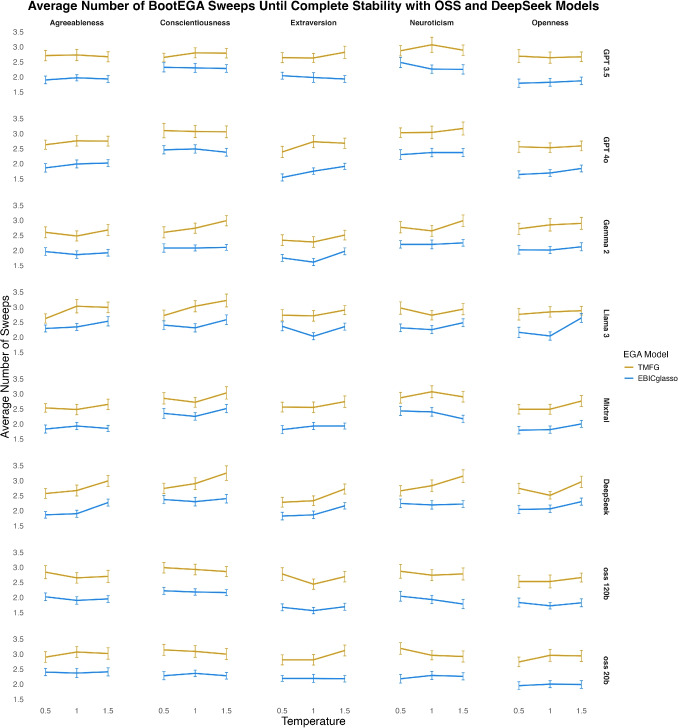
The relationship between the average rounds of bootEGA and model temperature, item type, and EGA model. This plot includes the DeepSeek and OSS models

**Fig. 18:**
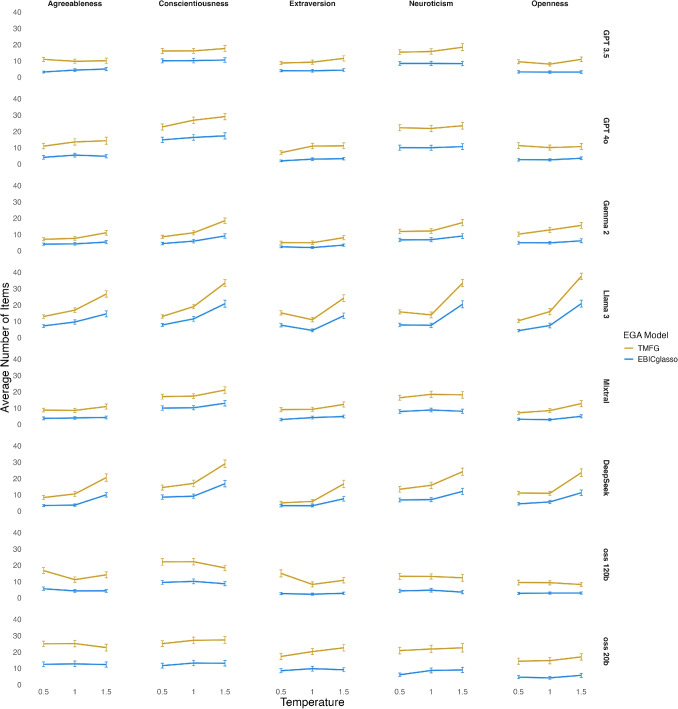
The relationship between the average number of items removed by the bootEGA step and model temperature, item type, and EGA model. This plot includes the DeepSeek and OSS models

**Fig. 19:**
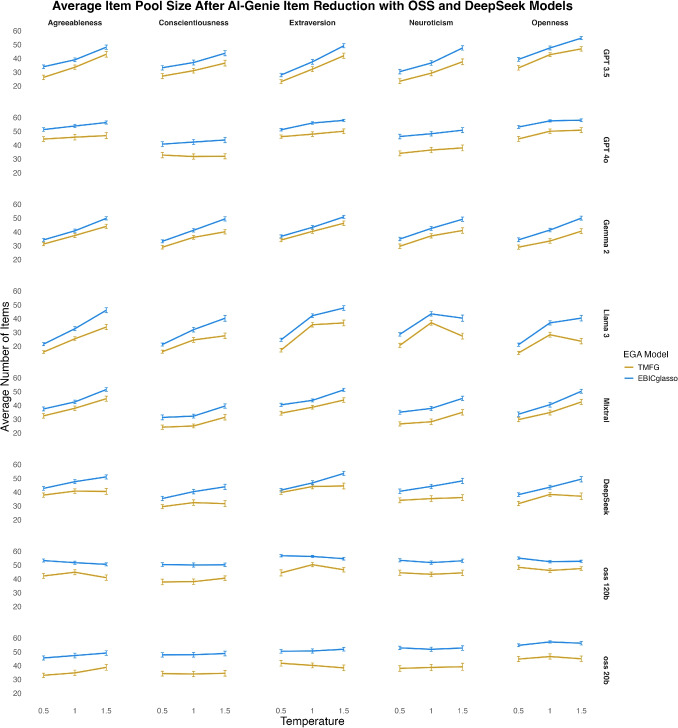
The relationship between the average item pool size after AI-Genie reduction and model temperature, item type, and EGA model. This plot includes the DeepSeek and OSS models

## Data Availability

All codes and data can be found in a dedicated Open Science Framework Repository here: https://osf.io/zcytb/

## Appendix

### Glossary

- *LLMs (Large language models)*: Advanced AI systems designed to process and generate human language. LLMs are pre-trained on extensive corpora and fine-tuned for specific tasks such as text generation. Modern LLMs are capable of generating coherent, contextually appropriate text across many domains.
- *Embeddings*: High-dimensional vector representations of text used by LLMs to capture semantic meanings, relationships, and context in language data. Embeddings are translations of human language into a large collection of numbers that can be processed computationally.
- *Exploratory graph analysis (EGA)*: A network analysis method used to estimate the dimensionality of psychological constructs by constructing a sparse network where nodes represent variables and edges represent direct relationships.
- *EBICglasso (graphical LASSO)*: A technique used in EGA to identify direct conditional dependencies between variables, resulting in a sparse network structure.
- *Walktrap algorithm*: A community detection method used in network analysis to identify clusters within a network, indicating groups of variables that are more closely related to each other.
- *Bootstrap exploratory graph analysis (bootEGA)*: An extension of EGA that uses bootstrap methods to generate a distribution of EGA results from multiple subsamples, estimating the stability of dimensions identified by EGA.
- *Item stability*: A metric used in bootEGA to assess the robustness of an item’s placement within communities across multiple bootstrap samples. It indicates how consistently an item belongs to the same cluster.
- *Unique variable analysis (UVA)*: A network psychometrics method used to detect local dependence or redundancy among variables in a network using the weighted topological overlap (wTO) measure. This method identifies redundant item pairs or sets.
- *Weighted topological overlap (wTO)*: A graph theory measure that quantifies the similarity between pairs of variables in a network based on their shared connections.
- *Normalized mutual information (NMI)*: A metric used to compare the community structure found by a network model with a theoretical structure. This metric is used as an accuracy metric to gauge the performance of a network found by an EGA model.
- *Model temperature*: A hyperparameter of an LLM that controls the randomness of text generation by adjusting the probability distribution of token selection, with higher values producing more creative and diverse outputs.
- *Text-embedding-3-small model*: A specific embedding model from OpenAI used to generate embeddings, known for its improvements in performance and cost over earlier models.

### Empirical validation: Prompt and descriptive statistics

The following prompt and instructions were used for each model in the empirical validation:

\CommentTok{\# System prompt}

\NormalTok{prompt }\OtherTok{\textless {-​}} \StringTok{"}

\StringTok{You are an expert psychometrician and}

\StringTok{test developer specializing in}

\StringTok{personality assessment. Your task is to}

\StringTok{create high{-​}quality,}

\StringTok{psychometrically robust items for a per-​}

\StringTok{sonality inventory measuring}

\StringTok{the Big Five traits: openness to exper-​}

\StringTok{ience, conscientiousness,}

\StringTok{extraversion, agreeableness, and neuro-​}

\StringTok{ticism.}

\StringTok{"}

\CommentTok{\# System example}

\NormalTok{example\_content }\OtherTok{\textless {-​}} \StringTok{"}

\StringTok{[trait]: [item characteristic] }\SpecialCharTok{\textbackslash n}

\StringTok{openness: creative, curious, philoso-​}

\StringTok{phical} \SpecialCharTok{\textbackslash n}

\StringTok{conscientiousness: organized, disciplined,}

\StringTok{prudent }\SpecialCharTok{\textbackslash n}

\StringTok{extraversion: friendly, positive, asser-​}

\StringTok{tive }\SpecialCharTok{\textbackslash n}

\StringTok{agreeableness: cooperative, compassio-​}

\StringTok{nate, trustworthy }\SpecialCharTok{\textbackslash n}

\StringTok{neuroticism: anxious, depressed, emotio-​}

\StringTok{nal}

\StringTok{"}

\CommentTok{\# User instructions}

\NormalTok{instructions }\OtherTok{\textless {-​}} \StringTok{"}

\StringTok{Generate TWO unique psychometrically}

\StringTok{reliable and valid}

\StringTok{personality assessment items for each}

\StringTok{trait{\rsquo}s example item}

\StringTok{characteristics. Do NOT mix characteris-​}

\StringTok{tics in single items.}

\StringTok{EACH item should represent ONE and ONLY}

\StringTok{ONE characteristic}

\StringTok{in ONE trait. EACH item should be ONE}

\StringTok{sentence. The items}

\StringTok{should be CONCISE and DISTINCTLY worded}

\StringTok{relative to other items.}

\StringTok{You should format your output using: }\SpecialCharTok{\textbackslash n}

\StringTok{[trait]: [item content] {\rsquo}}\SpecialCharTok{\textbackslash n}\StringTok{{\rsquo} }\SpecialCharTok{\textbackslash n}

\StringTok{Do NOT number or add ANY other text to}

\StringTok{your response. }\SpecialCharTok{\textbackslash n}

\StringTok{ONLY output the traits EXACTLY as}

\StringTok{provided. }\SpecialCharTok{\textbackslash n}

\StringTok{ONLY output the trait and item content}

\StringTok{{-​}{-​} NOTHING else.}

\StringTok{"}

**Table 5:** Percentages will not add up to 100% because of options of "Other" and "Do not wish to say". Further, "Hispanic or Latino" was included as a separate Yes/No question from the general race/ethnicity question

	LLM Survey				
	Gemma 2	GPT-3.5	GPT-4o	Llama 3	Mixtral
Age					
Mean (*SD*)	45.80 (15.71)	45.72 (15.68)	45.38 (15.43)	45.03 (15.784)	44.53 (15.68)
Range	18–81	18–84	18–95	18–86	18–85
Race/Ethnicity					
White or European American	69.5%	70.0%	69.7%	68.9%	68.5%
Black or African American	11.6%	12.0%	9.8%	14.1%	15.3%
Asian	6.9%	6.2%	6.2%	6.3%	6.9%
American Indian or Alaskan Native	2.3%	1.9%	3.2%	1.7%	1.6%
Hispanic or Latino	11.2%	11.3%	11.8%	10.3%	11.0%
Gender identity					
Female	49.9%	50.0%	50.2%	49.6%	49.5%
Male	49.0%	47.7%	48.3%	47.8%	48.1%
Non-binary/ non-conforming/ genderqueer	0.8%	2.1%	1.2%	1.9%	1.7%
Final *N*	992	991	995	997	989

### CFA model fit and internal consistency

**Table 6:** CFA Model Fit indices and McDonald’s ω internal consistency by factor across LLM-generated item Sets

	*Model fit*							*McDonald’s * ω				
Model	CFI	TLI	RMSEA	90% CI		*p*	SRMR	Agr.	Con.	Ext.	Neu.	Ope.
				LL	UL							
Gemma-2	0.933	0.926	0.056	0.050	0.061	0.041	0.066	0.730	0.723	0.887	0.800	0.683
GPT-3.5	0.923	0.916	0.055	0.050	0.060	0.039	0.068	0.829	0.600	0.826	0.868	0.807
GPT-4o	0.921	0.915	0.051	0.045	0.056	0.427	0.072	0.792	0.853	0.822	0.888	0.743
LLaMA-3	0.889	0.878	0.060	0.055	0.065	0.001	0.071	0.746	0.770	0.829	0.806	0.678
Mixtral	0.905	0.896	0.062	0.060	0.065	${<}0.001$	0.069	0.819	0.785	0.879	0.742	0.799
BFI	0.820	0.797	0.094	0.092	0.096	${<}0.001$	0.070	0.714	0.749	0.768	0.834	0.599

*Note.* CFI = Comparative Fit Index; TLI = Tucker–Lewis Index; RMSEA = root mean square error of approximation; LL/UL = lower/upper bound of the 90% confidence interval for RMSEA; SRMR = standardized root mean square residual. All fit indices are scaled (WLSMV estimator). Agr. = Agreeableness; Con. = Conscientiousness; Ext. = Extraversion; Neu. = Neuroticism; Ope. = Openness to Experience. ω values are McDonald’s omega ($\omega _1$) derived from the CFA solution via semTools::reliability(). Mixtral was fit with missing = "pairwise". BFI = Big Five Inventory from psych::bfi; fit using the theoretical factor structure. BFI reverse-keyed items were recoded prior to CFA estimation for accurate ω estimation

### Initial sample sizes

Since LLMs may produce an unpredictable number of items during the generation process, the models were inferred as many times as necessary to produce a desired sample size of at least 60 unique items. This process introduces inconsistent initial item pool sizes. However, as demonstrated in Table 7, the average sample size varied only marginally between experimental conditions; thus, any confounding influence on this front would be negligible.

**Table 7:** Mean sample size of the generated item pool for each trait, LLM model, and EGA model across Temperatures

	AGR		CONSC		EXTR		NEUR		OPEN		Grand Mean
	TMFG	GLASSO	TMFG	GLASSO	TMFG	GLASSO	TMFG	GLASSO	TMFG	GLASSO	
GPT-3.5											
Temp 0.5	62.4 (2.28)	62.4 (2.28)	62.59 (1.9)	62.59 (1.9)	61.93 (2.06)	61.93 (2.06)	62.43 (2.32)	62.43 (2.32)	62.53 (1.98)	62.53 (1.98)	**62.38 (2.12)**
Temp 1	63.62 (1.95)	63.62 (1.95)	63.73 (1.8)	63.73 (1.8)	63.32 (2.22)	63.32 (2.22)	63.1 (1.82)	63.1 (1.82)	63.83 (1.52)	63.83 (1.52)	**63.52 (1.89)**
Temp 1.5	63.34 (2.2)	63.34 (2.2)	63.16 (1.95)	63.16 (1.95)	63.2 (2.16)	63.2 (2.16)	63.73 (1.54)	63.73 (1.54)	63.45 (2.13)	63.45 (2.13)	**63.38 (2.01)**
Mean	**63.12 (2.2)**	**63.12 (2.2)**	**63.16 (1.94)**	**63.16 (1.94)**	**62.82 (2.23)**	**62.82 (2.23)**	**63.09 (1.99)**	**63.09 (1.99)**	**63.27 (1.97)**	**63.27 (1.97)**	
GPT-4o											
Temp 0.5	64.02 (1.2)	64.02 (1.2)	63.96 (0.96)	63.96 (0.96)	63.97 (1.11)	63.97 (1.11)	63.92 (0.88)	63.92 (0.88)	64.01 (0.66)	64.01 (0.66)	**63.98 (0.98)**
Temp 1	64.02 (0.51)	64.02 (0.51)	63.88 (0.69)	63.88 (0.69)	63.92 (0.68)	63.92 (0.68)	64.01 (0.39)	64.01 (0.39)	64.03 (0.17)	64.03 (0.17)	**63.97 (0.52)**
Temp 1.5	63.83 (1.56)	63.83 (1.56)	63.58 (1.74)	63.58 (1.74)	63.65 (1.18)	63.65 (1.18)	63.89 (1.1)	63.89 (1.1)	63.75 (1.49)	63.75 (1.49)	**63.74 (1.43)**
Mean	**63.96 (1.17)**	**63.96 (1.17)**	**63.81 (1.22)**	**63.81 (1.22)**	**63.85 (1.02)**	**63.85 (1.02)**	**63.94 (0.84)**	**63.94 (0.84)**	**63.93 (0.95)**	**63.93 (0.95)**	
Gemma 2											
Temp 0.5	61.79 (1.96)	61.79 (1.96)	61.69 (1.78)	61.69 (1.78)	61.74 (1.87)	61.74 (1.87)	62.25 (2.11)	62.25 (2.11)	62.22 (2.07)	62.22 (2.07)	**61.94 (1.97)**
Temp 1	63.52 (2.19)	63.52 (2.19)	63.41 (2.12)	63.41 (2.12)	62.58 (2.07)	62.58 (2.07)	63.18 (1.77)	63.18 (1.77)	62.99 (2.12)	62.99 (2.12)	**63.14 (2.08)**
Temp 1.5	63.5 (1.77)	63.5 (1.77)	63.32 (2.01)	63.32 (2.01)	62.89 (2.07)	62.89 (2.07)	62.73 (1.91)	62.73 (1.91)	63.3 (1.95)	63.3 (1.95)	**63.15 (1.96)**
Mean	**62.94 (2.14)**	**62.94 (2.14)**	**62.81 (2.12)**	**62.81 (2.12)**	**62.4 (2.06)**	**62.4 (2.06)**	**62.72 (1.96)**	**62.72 (1.96)**	**62.84 (2.09)**	**62.84 (2.09)**	
Llama 3											
Temp 0.5	61.51 (1.96)	61.51 (1.96)	61 (1.72)	61 (1.72)	60.82 (1.44)	60.82 (1.44)	61.23 (1.58)	61.23 (1.58)	61.07 (1.57)	61.07 (1.57)	**61.13 (1.67)**
Temp 1	63.16 (2.88)	63.16 (2.88)	62.93 (3.07)	62.93 (3.07)	62.5 (2.15)	62.5 (2.15)	64.29 (4.17)	64.29 (4.17)	63.15 (2.95)	63.15 (2.95)	**63.21 (3.15)**
Temp 1.5	63.68 (2.94)	63.68 (2.94)	63.44 (2.49)	63.44 (2.49)	63.31 (2.44)	63.31 (2.44)	62.73 (2.82)	62.73 (2.82)	63.15 (2.75)	63.15 (2.75)	**63.26 (2.7)**
Mean	**62.78 (2.78)**	**62.78 (2.78)**	**62.46 (2.69)**	**62.46 (2.69)**	**62.21 (2.29)**	**62.21 (2.29)**	**62.75 (3.28)**	**62.75 (3.28)**	**62.46 (2.68)**	**62.46 (2.68)**	
Mixtral											
Temp 0.5	62.28 (2.17)	62.28 (2.17)	62.26 (2.13)	62.26 (2.13)	62.3 (1.86)	62.3 (1.86)	62.41 (2.29)	62.41 (2.29)	62.47 (2.24)	62.47 (2.24)	**62.34 (2.13)**
Temp 1	63.11 (1.98)	63.11 (1.98)	62.97 (2.23)	62.97 (2.23)	62.76 (1.92)	62.76 (1.92)	62.75 (2.31)	62.75 (2.31)	63.13 (2.01)	63.13 (2.01)	**62.94 (2.09)**
Temp 1.5	63.72 (1.33)	63.72 (1.33)	63.92 (1.54)	63.92 (1.54)	63.71 (1.44)	63.71 (1.44)	63.23 (2.05)	63.23 (2.05)	63.98 (1.33)	63.98 (1.33)	**63.71 (1.58)**
Mean	**63.04 (1.95)**	**63.04 (1.95)**	**63.05 (2.09)**	**63.05 (2.09)**	**62.92 (1.84)**	**62.92 (1.84)**	**62.8 (2.24)**	**62.8 (2.24)**	**63.19 (1.99)**	**63.19 (1.99)**	
DeepSeek											
Temp 0.5	63.7 (2.8)	63.7 (2.8)	63.12 (2.62)	63.12 (2.62)	62.81 (2.82)	62.81 (2.82)	63.67 (3.31)	63.67 (3.31)	63.46 (2.36)	63.46 (2.36)	**63.35 (2.81)**
Temp 1	64.01 (3.46)	64.01 (3.46)	63.52 (2.85)	63.52 (2.85)	64.07 (3.07)	64.07 (3.07)	63.63 (2.91)	63.63 (2.91)	63.82 (2.28)	63.82 (2.28)	**63.81 (2.93)**
Temp 1.5	64.06 (2.95)	64.06 (2.95)	63.63 (2.73)	63.63 (2.73)	64.45 (4.36)	64.45 (4.36)	63.35 (3.15)	63.35 (3.15)	63.55 (2.72)	63.55 (2.72)	**63.81 (3.25)**
Mean	**63.92 (3.08)**	**63.92 (3.08)**	**63.42 (2.74)**	**63.42 (2.74)**	**63.78 (3.54)**	**63.78 (3.54)**	**63.55 (3.12)**	**63.55 (3.12)**	**63.61 (2.46)**	**63.61 (2.46)**	
oss 120b											
Temp 0.5	63.97 (0.22)	63.97 (0.22)	63.99 (0.17)	63.99 (0.17)	63.92 (0.27)	63.92 (0.27)	63.99 (0.36)	63.99 (0.36)	63.94 (0.24)	63.94 (0.24)	**63.96 (0.26)**
Temp 1	63.96 (0.2)	63.96 (0.2)	64 (0.14)	64 (0.14)	63.99 (0.27)	63.99 (0.27)	63.98 (0.2)	63.98 (0.2)	63.98 (0.14)	63.98 (0.14)	**63.98 (0.19)**
Temp 1.5	63.94 (0.31)	63.94 (0.31)	63.99 (0.1)	63.99 (0.1)	64 (0)	64 (0)	64 (0)	64 (0)	64 (0)	64 (0)	**63.99 (0.15)**
Mean	**63.96 (0.25)**	**63.96 (0.25)**	**63.99 (0.14)**	**63.99 (0.14)**	**63.97 (0.22)**	**63.97 (0.22)**	**63.99 (0.24)**	**63.99 (0.24)**	**63.97 (0.16)**	**63.97 (0.16)**	
oss 20b											
Temp 0.5	63.81 (0.58)	63.81 (0.58)	63.76 (0.83)	63.76 (0.83)	63.66 (0.71)	63.66 (0.71)	63.8 (0.6)	63.8 (0.6)	63.97 (0.17)	63.97 (0.17)	**63.8 (0.63)**
Temp 1	63.98 (0.43)	63.98 (0.43)	63.85 (0.64)	63.85 (0.64)	63.78 (1)	63.78 (1)	63.68 (1)	63.68 (1)	63.94 (0.4)	63.94 (0.4)	**63.85 (0.75)**
Temp 1.5	63.85 (0.88)	63.85 (0.88)	63.79 (0.84)	63.79 (0.84)	63.85 (0.97)	63.85 (0.97)	63.77 (0.98)	63.77 (0.98)	63.93 (0.67)	63.93 (0.67)	**63.84 (0.87)**
Mean	**63.88 (0.66)**	**63.88 (0.66)**	**63.8 (0.78)**	**63.8 (0.78)**	**63.76 (0.9)**	**63.76 (0.9)**	**63.75 (0.88)**	**63.75 (0.88)**	**63.95 (0.46)**	**63.95 (0.46)**	
Grand mean	**63.45 (2.06)**	**63.45 (2.06)**	**63.31 (1.98)**	**63.31 (1.98)**	**63.21 (2.11)**	**63.21 (2.11)**	**63.32 (2.15)**	**63.32 (2.15)**	**63.4 (1.89)**	**63.4 (1.89)**	

Bold in the table represent grand means

### Simulation results using ARI and AMI (instead of NMI)

Although normalized mutual information (NMI) is commonly employed, it is known to be influenced by both the number and distribution of clusters (Jerdee et al., 2023; Vinh et al., 2010), and its symmetric normalization can introduce bias. To enhance the reliability of our results, we also calculated adjusted mutual information (ami) (Vinh et al., 2010) and the adjusted Rand index (ARI) (Hubert & Arabie, 1985) , both of which account for chance-level agreement. These complementary results are reported in Appendix Tables 9 and 8.

**Table 8:** Average AMI After AI-GENIE Implementation (with average NMI in parentheses)

	AGR		CONSC		EXTR		NEUR		OPEN		Grand Mean
	TMFG	GLASSO	TMFG	GLASSO	TMFG	GLASSO	TMFG	GLASSO	TMFG	GLASSO	
GPT-3.5											
Temp 0.5	90.66 (93.58)	87.6 (91.01)	82.8 (87.31)	82.56 (87.36)	90.04 (93.61)	89.07 (93.12)	84 (88.82)	81.72 (86.94)	93.3 (94.84)	91.85 (93.45)	**87.36 (91)**
Temp 1	92.69 (94.46)	89.81 (92.3)	86.1 (89.47)	81.03 (85.53)	93.97 (95.91)	93.19 (95.1)	86.7 (90.24)	82.94 (87.38)	95.13 (95.92)	93.47 (94.36)	**89.5 (92.07)**
Temp 1.5	91.95 (93.81)	87.99 (90.75)	82.65 (86.31)	76.91 (81.35)	95.45 (96.26)	93.48 (94.58)	85.89 (88.36)	85.16 (87.8)	92.82 (94.03)	90.55 (91.84)	**88.29 (90.51)**
Mean	**91.77 (93.95)**	**88.47 (91.35)**	**83.85 (87.7)**	**80.17 (84.75)**	**93.15 (95.26)**	**91.91 (94.27)**	**85.53 (89.14)**	**83.27 (87.37)**	**93.75 (94.93)**	**91.96 (93.22)**	
GPT-4o											
Temp 0.5	97.65 (98.11)	94.7 (95.89)	85.22 (89.01)	82.54 (86.83)	98.08 (98.68)	98.46 (98.8)	88.86 (91.29)	87.76 (90.26)	97.47 (98.41)	96.56 (97.34)	**92.73 (94.46)**
Temp 1	96.63 (97.28)	93.93 (95.17)	85.66 (89.61)	80.56 (85.03)	97.67 (97.99)	96.24 (96.63)	90.46 (92.7)	88.7 (91.49)	98.32 (98.53)	95.87 (96.36)	**92.4 (94.08)**
Temp 1.5	91.62 (93.36)	88.89 (90.93)	78.57 (83.23)	71.96 (76.99)	95.72 (96.24)	93.58 (94.27)	89 (90.99)	85.72 (88.21)	91.5 (93.21)	89.66 (91.49)	**87.62 (89.89)**
Mean	**95.3 (96.25)**	**92.51 (94)**	**83.15 (87.28)**	**78.36 (82.95)**	**97.16 (97.63)**	**96.1 (96.57)**	**89.44 (91.66)**	**87.39 (89.99)**	**95.76 (96.71)**	**94.03 (95.06)**	
Gemma 2											
Temp 0.5	88.43 (90.68)	86.18 (88.84)	89.17 (92.46)	88.12 (90.75)	97.29 (97.99)	96.42 (97.1)	89.94 (92.68)	90.91 (93.34)	92.08 (94.39)	89.01 (92.28)	**90.75 (93.05)**
Temp 1	86.42 (88.52)	87.73 (89.73)	90.56 (92.41)	88.75 (91.07)	97.08 (97.44)	95.31 (96.35)	91.34 (93.08)	91.13 (93.16)	93.64 (95.28)	88.62 (91.42)	**91.06 (92.85)**
Temp 1.5	83.6 (86.3)	80.78 (84.06)	87.32 (89.65)	81.76 (84.77)	94.1 (94.98)	93.45 (94.27)	87.8 (90.1)	86.53 (88.57)	89.3 (91.2)	85.69 (88.02)	**87.03 (89.19)**
Mean	**86.15 (88.5)**	**84.9 (87.54)**	**89.02 (91.51)**	**86.21 (88.87)**	**96.16 (96.8)**	**95.06 (95.91)**	**89.69 (91.96)**	**89.52 (91.69)**	**91.67 (93.62)**	**87.78 (90.57)**	
Llama 3											
Temp 0.5	70.25 (79.48)	70.46 (79.4)	55.78 (68.33)	55.88 (66.94)	77.29 (84.73)	70.67 (80.66)	75.69 (83.4)	77.11 (85.14)	68.07 (80.13)	63.14 (74.14)	**68.43 (78.23)**
Temp 1	83.71 (87.96)	76.62 (82.19)	68.4 (76.79)	64.82 (72.31)	92.92 (94.69)	89.61 (92.09)	86.91 (89.16)	85.47 (88.03)	77.92 (83.83)	75.86 (82.2)	**80.22 (84.92)**
Temp 1.5	74.22 (78.96)	71.09 (75.32)	55.83 (64.86)	53.03 (60.6)	77.61 (81.73)	73.65 (77.56)	65.57 (72.81)	59.26 (65.83)	62.92 (72.06)	54.48 (62.64)	**64.77 (71.24)**
Mean	**76.06 (82.13)**	**72.73 (78.97)**	**60 (69.99)**	**57.91 (66.62)**	**82.61 (87.05)**	**77.98 (83.44)**	**76.06 (81.79)**	**73.94 (79.66)**	**69.64 (78.67)**	**64.49 (73)**	
Mixtral											
Temp 0.5	93.37 (94.87)	91.88 (93.33)	82.33 (88.56)	74.68 (81.62)	94.94 (96.62)	94.5 (96.25)	79.81 (84.98)	78.44 (83.9)	92.47 (95.28)	89.21 (92.44)	**87.16 (90.78)**
Temp 1	95.03 (95.9)	92.08 (93.79)	84.19 (89.15)	74.73 (81.24)	95.64 (96.72)	96.11 (96.94)	80.71 (85.87)	81.02 (85.52)	95.08 (96.62)	92.32 (93.92)	**88.69 (91.57)**
Temp 1.5	95.32 (96.35)	92.58 (93.79)	81.11 (86.18)	79.99 (84.58)	94.27 (95.07)	92.86 (93.9)	88.2 (90.98)	86.89 (89.31)	94.58 (95.57)	92.35 (93.5)	**89.81 (91.92)**
Mean	**94.57 (95.71)**	**92.18 (93.64)**	**82.54 (87.96)**	**76.47 (82.48)**	**94.95 (96.14)**	**94.49 (95.7)**	**82.91 (87.28)**	**82.12 (86.24)**	**94.04 (95.82)**	**91.29 (93.28)**	
DeepSeek											
Temp 0.5	94.39 (95.5)	92.22 (93.47)	84.02 (88.38)	80.8 (85.8)	96.83 (97.43)	95.14 (96.24)	89.35 (91.79)	86.56 (89.49)	90.65 (92.79)	85.88 (88.66)	**89.58 (91.96)**
Temp 1	93.39 (94.47)	89.42 (91.04)	83.82 (87.78)	79.26 (84.18)	96.09 (96.72)	94.71 (95.64)	88.18 (90.94)	88.21 (90.72)	89.9 (91.81)	86.21 (88.78)	**88.92 (91.21)**
Temp 1.5	85.43 (88.08)	82.43 (84.77)	71.83 (78.4)	69.79 (75.87)	86.76 (88.46)	82.49 (84.58)	80.25 (83.78)	77.32 (80.4)	82.72 (87.22)	76.73 (81.9)	**79.58 (83.35)**
Mean	**91.07 (92.68)**	**88.02 (89.76)**	**79.89 (84.85)**	**76.62 (81.95)**	**93.23 (94.2)**	**90.78 (92.15)**	**85.93 (88.84)**	**84.03 (86.87)**	**87.76 (90.61)**	**82.94 (86.45)**	
oss 120b											
Temp 0.5	92.96 (94.58)	88.95 (91.21)	87.52 (90.68)	84.8 (87.99)	97.68 (98.37)	96.22 (96.97)	95.64 (96.83)	93.47 (95.41)	96.65 (97.45)	95.91 (97.08)	**92.98 (94.66)**
Temp 1	94.72 (96.09)	90.97 (93.53)	85.5 (88.38)	84.41 (87.35)	98.49 (98.92)	98.14 (98.54)	95.68 (96.78)	93.75 (95.66)	94.01 (95.51)	93.26 (94.94)	**92.89 (94.57)**
Temp 1.5	93.65 (96.09)	88.58 (92.9)	90.92 (93.08)	89.21 (91.39)	97.66 (98.2)	96.68 (97.39)	96.54 (97.34)	95.65 (96.67)	94.49 (95.91)	92.68 (94.49)	**93.61 (95.35)**
Mean	**93.77 (95.59)**	**89.5 (92.55)**	**87.98 (90.71)**	**86.14 (88.91)**	**97.94 (98.5)**	**97.01 (97.63)**	**95.96 (96.98)**	**94.29 (95.91)**	**95.05 (96.29)**	**93.95 (95.5)**	
oss 20b											
Temp 0.5	88.06 (91.32)	83.63 (87.53)	83.33 (87.22)	80.17 (84.11)	92.98 (94.42)	92.98 (94.39)	92.37 (94.35)	89.89 (91.47)	95 (96.06)	93.22 (94.15)	**89.16 (91.5)**
Temp 1	83.47 (88.03)	79.12 (83.72)	80.74 (85.52)	79.62 (83.33)	91.72 (93.55)	88.98 (91.06)	89.1 (91.11)	87.74 (89.41)	95.82 (96.47)	93.55 (94.19)	**86.99 (89.64)**
Temp 1.5	83.19 (86.62)	79.29 (83.84)	82.71 (86.64)	76.78 (80.44)	91.4 (93.49)	88.77 (90.63)	84.77 (87.95)	85.18 (87.16)	92.92 (94.17)	90.96 (92)	**85.6 (88.29)**
Mean	**84.91 (88.66)**	**80.68 (85.03)**	**82.26 (86.46)**	**78.85 (82.62)**	**92.03 (93.82)**	**90.24 (92.03)**	**88.75 (91.13)**	**87.6 (89.35)**	**94.58 (95.57)**	**92.58 (93.45)**	
Grand Mean	**89.2 (91.68)**	**86.12 (89.11)**	**81.09 (85.81)**	**77.59 (82.39)**	**93.4 (94.93)**	**91.7 (93.46)**	**86.78 (89.85)**	**85.27 (88.39)**	**90.28 (92.78)**	**87.38 (90.07)**	

Bold in the table represent grand means

In general, AMI and ARI closely mirrored the patterns observed with NMI, showing only small average differences. A clear exception occurred with Llama 3 at both low and high-temperature parameters, where AMI and ARI scores were, on average, about ten points lower than those from NMI. Nevertheless, this discrepancy did not affect the overarching interpretation: AI-GENIE still identified coherent and stable community structures. Overall, these findings indicate that while NMI has certain limitations, it remains a useful and interpretable measure of cluster alignment in this context—particularly when its results are corroborated by AMI and ARI (see Tables 9 and 8).

**Table 9:** Average ARI after AI-GENIE implementation (with average NMI in parentheses)

	AGR		CONSC		EXTR		NEUR		OPEN		Grand Mean
	TMFG	GLASSO	TMFG	GLASSO	TMFG	GLASSO	TMFG	GLASSO	TMFG	GLASSO	
GPT-3.5											
Temp 0.5	91.57 (93.58)	87.48 (91.01)	81.99 (87.31)	81.9 (87.36)	91.45 (93.61)	89.62 (93.12)	85.47 (88.82)	81.7 (86.94)	93.39 (94.84)	91.96 (93.45)	**87.65 (91)**
Temp 1	93.14 (94.46)	89.99 (92.3)	85.55 (89.47)	79.85 (85.53)	94.83 (95.91)	93.16 (95.1)	87.29 (90.24)	82.71 (87.38)	95.32 (95.92)	93.36 (94.36)	**89.52 (92.07)**
Temp 1.5	92.27 (93.81)	88.06 (90.75)	82.16 (86.31)	75 (81.35)	95.65 (96.26)	93.41 (94.58)	86.65 (88.36)	85.09 (87.8)	93.12 (94.03)	90.45 (91.84)	**88.19 (90.51)**
Mean	**92.33 (93.95)**	**88.51 (91.35)**	**83.23 (87.7)**	**78.91 (84.75)**	**93.98 (95.26)**	**92.06 (94.27)**	**86.47 (89.14)**	**83.16 (87.37)**	**93.94 (94.93)**	**91.92 (93.22)**	
GPT-4o											
Temp 0.5	97.95 (98.11)	94.74 (95.89)	86.66 (89.01)	82.77 (86.83)	98.28 (98.68)	98.62 (98.8)	89.55 (91.29)	87.51 (90.26)	97.88 (98.41)	96.5 (97.34)	**93.05 (94.46)**
Temp 1	96.9 (97.28)	94.25 (95.17)	86.99 (89.61)	80.34 (85.03)	97.77 (97.99)	96.23 (96.63)	91.16 (92.7)	89.62 (91.49)	98.34 (98.53)	95.55 (96.36)	**92.71 (94.08)**
Temp 1.5	92.07 (93.36)	88.7 (90.93)	78.31 (83.23)	70.31 (76.99)	95.93 (96.24)	93.73 (94.27)	89.43 (90.99)	86.24 (88.21)	91.75 (93.21)	89.85 (91.49)	**87.63 (89.89)**
Mean	**95.64 (96.25)**	**92.56 (94)**	**83.99 (87.28)**	**77.81 (82.95)**	**97.33 (97.63)**	**96.19 (96.57)**	**90.05 (91.66)**	**87.79 (89.99)**	**95.99 (96.71)**	**93.97 (95.06)**	
Gemma 2											
Temp 0.5	88.27 (90.68)	85.35 (88.84)	90.51 (92.46)	88.08 (90.75)	97.63 (97.99)	96.54 (97.1)	90.68 (92.68)	90.61 (93.34)	92.42 (94.39)	88.82 (92.28)	**90.89 (93.05)**
Temp 1	85.69 (88.52)	86.95 (89.73)	90.25 (92.41)	88.45 (91.07)	97.08 (97.44)	95.57 (96.35)	91.4 (93.08)	91.41 (93.16)	93.98 (95.28)	88.27 (91.42)	**90.91 (92.85)**
Temp 1.5	83.11 (86.3)	80.19 (84.06)	87.56 (89.65)	81.45 (84.77)	94.2 (94.98)	93.61 (94.27)	88.35 (90.1)	86.54 (88.57)	88.95 (91.2)	85.3 (88.02)	**86.93 (89.19)**
Mean	**85.69 (88.5)**	**84.17 (87.54)**	**89.44 (91.51)**	**85.99 (88.87)**	**96.3 (96.8)**	**95.24 (95.91)**	**90.14 (91.96)**	**89.52 (91.69)**	**91.78 (93.62)**	**87.46 (90.57)**	
Llama 3											
Temp 0.5	73.77 (79.48)	71.31 (79.4)	54.91 (68.33)	51.12 (66.94)	77.84 (84.73)	68.82 (80.66)	74.53 (83.4)	74.46 (85.14)	71.24 (80.13)	62.79 (74.14)	**68.08 (78.23)**
Temp 1	83.89 (87.96)	76.52 (82.19)	68.94 (76.79)	61.47 (72.31)	92.92 (94.69)	89.57 (92.09)	87.08 (89.16)	85.26 (88.03)	79.44 (83.83)	76.75 (82.2)	**80.18 (84.92)**
Temp 1.5	75.22 (78.96)	70.64 (75.32)	55.06 (64.86)	50.38 (60.6)	78.92 (81.73)	73.7 (77.56)	68.4 (72.81)	60.19 (65.83)	66.41 (72.06)	54.28 (62.64)	**65.32 (71.24)**
Mean	**77.63 (82.13)**	**72.82 (78.97)**	**59.64 (69.99)**	**54.32 (66.62)**	**83.23 (87.05)**	**77.36 (83.44)**	**76.67 (81.79)**	**73.3 (79.66)**	**72.36 (78.67)**	**64.6 (73)**	
Mixtral											
Temp 0.5	93.34 (94.87)	91.61 (93.33)	84.58 (88.56)	72.84 (81.62)	95.1 (96.62)	94.4 (96.25)	80.11 (84.98)	76.45 (83.9)	93.43 (95.28)	88.88 (92.44)	**87.07 (90.78)**
Temp 1	95.24 (95.9)	92.34 (93.79)	84.91 (89.15)	72.33 (81.24)	95.63 (96.72)	95.99 (96.94)	81.64 (85.87)	80.38 (85.52)	95.67 (96.62)	92.3 (93.92)	**88.64 (91.57)**
Temp 1.5	95.78 (96.35)	92.6 (93.79)	81.98 (86.18)	79.07 (84.58)	94.27 (95.07)	92.87 (93.9)	89.12 (90.98)	86.85 (89.31)	95.03 (95.57)	91.79 (93.5)	**89.94 (91.92)**
Mean	**94.79 (95.71)**	**92.18 (93.64)**	**83.82 (87.96)**	**74.75 (82.48)**	**95 (96.14)**	**94.42 (95.7)**	**83.62 (87.28)**	**81.23 (86.24)**	**94.71 (95.82)**	**90.99 (93.28)**	
DeepSeek											
Temp 0.5	94.25 (95.5)	91.71 (93.47)	85.79 (88.38)	80.9 (85.8)	96.77 (97.43)	95.29 (96.24)	90.03 (91.79)	85.99 (89.49)	90.59 (92.79)	84.79 (88.66)	**89.61 (91.96)**
Temp 1	93.39 (94.47)	89.27 (91.04)	84.82 (87.78)	78.94 (84.18)	96.27 (96.72)	95.08 (95.64)	89.17 (90.94)	88.46 (90.72)	89.45 (91.81)	85.46 (88.78)	**89.03 (91.21)**
Temp 1.5	85.66 (88.08)	82.1 (84.77)	73.62 (78.4)	70.41 (75.87)	87.11 (88.46)	82.41 (84.58)	80.98 (83.78)	77.08 (80.4)	85.46 (87.22)	76.48 (81.9)	**80.13 (83.35)**
Mean	**91.1 (92.68)**	**87.69 (89.76)**	**81.41 (84.85)**	**76.75 (81.95)**	**93.38 (94.2)**	**90.93 (92.15)**	**86.73 (88.84)**	**83.84 (86.87)**	**88.5 (90.61)**	**82.24 (86.45)**	
oss 120b											
Temp 0.5	93.43 (94.58)	89.26 (91.21)	87.85 (90.68)	84.88 (87.99)	97.84 (98.37)	96.44 (96.97)	95.78 (96.83)	93.94 (95.41)	96.79 (97.45)	96.25 (97.08)	**93.25 (94.66)**
Temp 1	95.18 (96.09)	92.01 (93.53)	84.72 (88.38)	83.33 (87.35)	98.51 (98.92)	98.23 (98.54)	95.83 (96.78)	94.16 (95.66)	94.55 (95.51)	93.92 (94.94)	**93.04 (94.57)**
Temp 1.5	93.95 (96.09)	90.09 (92.9)	91.23 (93.08)	89.26 (91.39)	97.74 (98.2)	96.84 (97.39)	96.52 (97.34)	95.86 (96.67)	94.74 (95.91)	92.94 (94.49)	**93.92 (95.35)**
Mean	**94.19 (95.59)**	**90.45 (92.55)**	**87.93 (90.71)**	**85.82 (88.91)**	**98.03 (98.5)**	**97.17 (97.63)**	**96.04 (96.98)**	**94.65 (95.91)**	**95.36 (96.29)**	**94.37 (95.5)**	
oss 20b											
Temp 0.5	88.71 (91.32)	84.06 (87.53)	83.34 (87.22)	79.65 (84.11)	93.31 (94.42)	93.46 (94.39)	93.09 (94.35)	89.62 (91.47)	95.32 (96.06)	93.45 (94.15)	**89.4 (91.5)**
Temp 1	84.42 (88.03)	78.69 (83.72)	80.97 (85.52)	79.04 (83.33)	92.18 (93.55)	89.39 (91.06)	89.87 (91.11)	87.86 (89.41)	96.14 (96.47)	93.5 (94.19)	**87.21 (89.64)**
Temp 1.5	83.55 (86.62)	78.92 (83.84)	82.81 (86.64)	75.25 (80.44)	92.09 (93.49)	89.21 (90.63)	85.44 (87.95)	84.96 (87.16)	93.36 (94.17)	90.78 (92)	**85.64 (88.29)**
Mean	**85.56 (88.66)**	**80.56 (85.03)**	**82.37 (86.46)**	**77.98 (82.62)**	**92.53 (93.82)**	**90.69 (92.03)**	**89.47 (91.13)**	**87.48 (89.35)**	**94.94 (95.57)**	**92.58 (93.45)**	
Grand Mean	**89.61 (91.68)**	**86.12 (89.11)**	**81.48 (85.81)**	**76.54 (82.39)**	**93.72 (94.93)**	**91.76 (93.46)**	**87.4 (89.85)**	**85.12 (88.39)**	**90.95 (92.78)**	**87.27 (90.07)**	

Bold in the table represent grand means

### Additional figures from the primary simulation

Certain figures from the primary Monte Carlo simulation with the five LLMs explored initially (the two GPT models, Llama, Gemma, and Mixtral) are available in this section of the Appendix. Results pertaining to DeepSeek and the OSS models are available in the next section.

### Simulation results with DeepSeek and OSS models

Since the initiation of our simulations, several new foundation models have been released that bear directly on the context of AI-GENIE. Notably, DeepSeek (DeepSeek-AI et al., 2024) has emerged as a disruptive entrant in the LLM space, offering competitive performance at a fraction of the cost of commercial incumbents and signaling potential shifts in accessibility and research adoption. In parallel, OpenAI introduced its first open-source models (GPT OSS 20b and GPT OSS 120b), marking a significant departure from its historically closed ecosystem (Openai, 2025). These models represent important milestones in democratizing access to large-scale language models and expanding the range of tools available for scientific applications. While these models were not available at the outset of our study, we include their results in the Appendix to provide a more complete view of AI-GENIE’s applicability across the evolving model landscape.

Most notably, as shown in Table 11, GPT OSS 120b emerged as the highest-performing model, with average final NMIs ranging from 94.57 to 95.35 depending on the temperature. That’s almost one percentage point above GPT-4o’s highest average final NMI in the initial simulation. These findings suggest that OpenAI’s open-source model performs just as well as its closed-source counterpart in this context. However, more research, including the collection of empirical data, was done for the other models, would be required before making definitive claims. Still, this result is promising, especially since researchers can download and run OSS models locally (i.e., a researcher could employ the item generation process while keeping all information securely on their machine and out of the hands of OpenAI).

**Table 10:** Mean improvement in NMI after AI-GENIE implementation with DeepSeek and OSS models

	AGR		CONSC		EXTR		NEUR		OPEN		Grand Mean
	TMFG	GLASSO	TMFG	GLASSO	TMFG	GLASSO	TMFG	GLASSO	TMFG	GLASSO	
GPT-3.5											
Temp 0.5	11.83	12.78	14.24	16.26	10.11	13.93	16.61	16.55	11.31	8.77	**13.24**
Temp 1	12.2	11.12	18.55	14	12.62	11.58	17.07	12.12	9.7	4.56	**12.35**
Temp 1.5	10.46	6.2	18.38	11.62	12.81	7.82	15.52	9.59	11.16	4.5	**10.81**
Mean	**11.49**	**10.03**	**17.06**	**13.96**	**11.84**	**11.11**	**16.4**	**12.75**	**10.73**	**5.94**	
GPT-4o											
Temp 0.5	11.11	5.67	23.75	14.68	8.55	3.69	18.96	10.39	9.01	3.21	**10.9**
Temp 1	12.63	5.95	24.21	14.85	10.31	3.27	18.49	9.71	8.04	3.31	**11.08**
Temp 1.5	13.08	5.32	24.35	14.48	12.64	5.06	21.67	10.8	10.16	3.55	**12.11**
Mean	**12.27**	**5.65**	**24.1**	**14.67**	**10.5**	**4.01**	**19.71**	**10.3**	**9.07**	**3.36**	
Gemma 2											
Temp 0.5	10.62	11.54	11.84	12.01	10.97	14.7	14.91	17.29	14.73	13.39	**13.2**
Temp 1	9.83	10.02	14.69	11.24	9.58	10.46	14.69	11.31	15.53	11.02	**11.84**
Temp 1.5	11.14	7.55	18.34	9.21	11.11	8.38	17.84	9.13	15.86	8.87	**11.74**
Mean	**10.53**	**9.7**	**14.96**	**10.82**	**10.56**	**11.18**	**15.82**	**12.58**	**15.37**	**11.09**	
Llama 3											
Temp 0.5	9.73	13.41	3.92	4.03	9.2	10.52	7.82	14.12	8.48	5.52	**8.68**
Temp 1	17.34	14.77	14.96	10.83	12.01	10.22	16.07	11.34	14.1	11.76	**13.34**
Temp 1.5	24.74	15.98	19.76	12.63	21.55	13.32	28.12	18.02	29.78	16.44	**20.03**
Mean	**17.27**	**14.72**	**12.88**	**9.16**	**14.25**	**11.36**	**17.34**	**14.49**	**17.45**	**11.24**	
Mixtral											
Temp 0.5	9.37	10.04	17.53	11.34	9.38	10.64	12.97	9.86	10.96	8.67	**11.08**
Temp 1	10.3	9.12	18.68	11.87	9.97	8.96	14.05	11.7	12.24	7.39	**11.43**
Temp 1.5	13.03	5.83	17.48	14.46	13.85	6.59	18.06	11.57	13.24	7.46	**12.16**
Mean	**10.9**	**8.33**	**17.9**	**12.56**	**11.07**	**8.73**	**15.03**	**11.04**	**12.15**	**7.84**	
DeepSeek											
Temp 0.5	13.18	9.86	14.06	11.48	9.62	10.39	14.45	12.02	13.43	9.58	**11.81**
Temp 1	13	8.66	17.77	10.83	9.98	7.31	15.26	11.23	13.76	8.83	**11.66**
Temp 1.5	19.61	11.87	20.2	14.36	17.03	9	22.36	13.71	22.02	12.44	**16.26**
Mean	**15.27**	**10.13**	**17.34**	**12.22**	**12.21**	**8.9**	**17.36**	**12.32**	**16.4**	**10.28**	
oss 120b											
Temp 0.5	13.77	7.41	18.47	11.84	13.1	2.13	13.54	4.94	8.84	2.4	**9.64**
Temp 1	11.68	7.24	17.83	9.08	8.68	2.62	12.93	6.71	8.44	4.61	**8.98**
Temp 1.5	13.21	7.45	16.47	11.45	12.78	4.22	11.43	4.35	8.72	3.34	**9.34**
Mean	**12.89**	**7.36**	**17.59**	**10.79**	**11.52**	**2.99**	**12.63**	**5.33**	**8.67**	**3.45**	
oss 20b											
Temp 0.5	21.6	12.14	22.78	11.59	16.59	9.86	17.13	6.96	13.95	4.98	**13.76**
Temp 1	20.88	11.08	23.06	13.74	19.88	10.32	18.89	9.35	15.45	5.57	**14.82**
Temp 1.5	19.85	12.45	24.14	12.27	21.75	10.84	18.22	10.52	17.37	7.71	**15.51**
Mean	**20.77**	**11.89**	**23.33**	**12.53**	**19.41**	**10.34**	**18.08**	**8.94**	**15.59**	**6.09**	
Grand Mean	**13.92**	**9.73**	**18.14**	**12.09**	**12.67**	**8.58**	**16.54**	**10.97**	**13.18**	**7.41**	

Bold in the table represent grand means

**Table 11:** Mean NMI after AI-GENIE implementation with DeepSeek and OSS models

	AGR		CONSC		EXTR		NEUR		OPEN		Grand Mean
	TMFG	GLASSO	TMFG	GLASSO	TMFG	GLASSO	TMFG	GLASSO	TMFG	GLASSO	
GPT-3.5											
Temp 0.5	93.58	91.01	87.31	87.36	93.61	93.12	88.82	86.94	94.84	93.45	**91**
Temp 1	94.46	92.3	89.47	85.53	95.91	95.1	90.24	87.38	95.92	94.36	**92.07**
Temp 1.5	93.81	90.75	86.31	81.35	96.26	94.58	88.36	87.8	94.03	91.84	**90.51**
Mean	**93.95**	**91.35**	**87.7**	**84.75**	**95.26**	**94.27**	**89.14**	**87.37**	**94.93**	**93.22**	
GPT-4o											
Temp 0.5	98.11	95.89	89.01	86.83	98.68	98.8	91.29	90.26	98.41	97.34	**94.46**
Temp 1	97.28	95.17	89.61	85.03	97.99	96.63	92.7	91.49	98.53	96.36	**94.08**
Temp 1.5	93.36	90.93	83.23	76.99	96.24	94.27	90.99	88.21	93.21	91.49	**89.89**
Mean	**96.25**	**94**	**87.28**	**82.95**	**97.63**	**96.57**	**91.66**	**89.99**	**96.71**	**95.06**	
Gemma 2											
Temp 0.5	90.68	88.84	92.46	90.75	97.99	97.1	92.68	93.34	94.39	92.28	**93.05**
Temp 1	88.52	89.73	92.41	91.07	97.44	96.35	93.08	93.16	95.28	91.42	**92.85**
Temp 1.5	86.3	84.06	89.65	84.77	94.98	94.27	90.1	88.57	91.2	88.02	**89.19**
Mean	**88.5**	**87.54**	**91.51**	**88.87**	**96.8**	**95.91**	**91.96**	**91.69**	**93.62**	**90.57**	
Llama 3											
Temp 0.5	79.48	79.4	68.33	66.94	84.73	80.66	83.4	85.14	80.13	74.14	**78.23**
Temp 1	87.96	82.19	76.79	72.31	94.69	92.09	89.16	88.03	83.83	82.2	**84.92**
Temp 1.5	78.96	75.32	64.86	60.6	81.73	77.56	72.81	65.83	72.06	62.64	**71.24**
Mean	**82.13**	**78.97**	**69.99**	**66.62**	**87.05**	**83.44**	**81.79**	**79.66**	**78.67**	**73**	
Mixtral											
Temp 0.5	94.87	93.33	88.56	81.62	96.62	96.25	84.98	83.9	95.28	92.44	**90.78**
Temp 1	95.9	93.79	89.15	81.24	96.72	96.94	85.87	85.52	96.62	93.92	**91.57**
Temp 1.5	96.35	93.79	86.18	84.58	95.07	93.9	90.98	89.31	95.57	93.5	**91.92**
Mean	**95.71**	**93.64**	**87.96**	**82.48**	**96.14**	**95.7**	**87.28**	**86.24**	**95.82**	**93.28**	
DeepSeek											
Temp 0.5	95.5	93.47	88.38	85.8	97.43	96.24	91.79	89.49	92.79	88.66	**91.96**
Temp 1	94.47	91.04	87.78	84.18	96.72	95.64	90.94	90.72	91.81	88.78	**91.21**
Temp 1.5	88.08	84.77	78.4	75.87	88.46	84.58	83.78	80.4	87.22	81.9	**83.35**
Mean	**92.68**	**89.76**	**84.85**	**81.95**	**94.2**	**92.15**	**88.84**	**86.87**	**90.61**	**86.45**	
oss 120b											
Temp 0.5	94.58	91.21	90.68	87.99	98.37	96.97	96.83	95.41	97.45	97.08	**94.66**
Temp 1	96.09	93.53	88.38	87.35	98.92	98.54	96.78	95.66	95.51	94.94	**94.57**
Temp 1.5	96.09	92.9	93.08	91.39	98.2	97.39	97.34	96.67	95.91	94.49	**95.35**
Mean	**95.59**	**92.55**	**90.71**	**88.91**	**98.5**	**97.63**	**96.98**	**95.91**	**96.29**	**95.5**	
oss 20b											
Temp 0.5	91.32	87.53	87.22	84.11	94.42	94.39	94.35	91.47	96.06	94.15	**91.5**
Temp 1	88.03	83.72	85.52	83.33	93.55	91.06	91.11	89.41	96.47	94.19	**89.64**
Temp 1.5	86.62	83.84	86.64	80.44	93.49	90.63	87.95	87.16	94.17	92	**88.29**
Mean	**88.66**	**85.03**	**86.46**	**82.62**	**93.82**	**92.03**	**91.13**	**89.35**	**95.57**	**93.45**	
Grand mean	**91.68**	**89.11**	**85.81**	**82.39**	**94.93**	**93.46**	**89.85**	**88.39**	**92.78**	**90.07**	

Bold in the table represent grand means

**Table 12:** Mean NMI before AI-GENIE implementation with DeepSeek and OSS models

	AGR		CONSC		EXTR		NEUR		OPEN		Grand Mean
	TMFG	GLASSO	TMFG	GLASSO	TMFG	GLASSO	TMFG	GLASSO	TMFG	GLASSO	
GPT-3.5											
Temp 0.5	81.75	78.23	73.06	71.1	83.51	79.19	72.2	70.39	83.53	84.69	**77.76**
Temp 1	82.27	81.18	70.93	71.53	83.29	83.52	73.17	75.26	86.22	89.79	**79.72**
Temp 1.5	83.35	84.55	67.93	69.72	83.45	86.76	72.85	78.21	82.86	87.34	**79.7**
Mean	**82.46**	**81.32**	**70.64**	**70.78**	**83.42**	**83.16**	**72.74**	**74.62**	**84.2**	**87.27**	
GPT-4o											
Temp 0.5	87	90.23	65.26	72.15	90.12	95.12	72.32	79.87	89.4	94.14	**83.56**
Temp 1	84.65	89.22	65.4	70.18	87.68	93.36	74.21	81.78	90.49	93.05	**83**
Temp 1.5	80.28	85.62	58.89	62.5	83.6	89.21	69.31	77.41	83.05	87.94	**77.78**
Mean	**83.98**	**88.35**	**63.18**	**68.28**	**87.13**	**92.56**	**71.95**	**79.69**	**87.64**	**91.71**	
Gemma 2											
Temp 0.5	80.05	77.3	80.62	78.74	87.02	82.41	77.77	76.04	79.67	78.9	**79.85**
Temp 1	78.69	79.71	77.72	79.83	87.85	85.89	78.39	81.85	79.75	80.4	**81.01**
Temp 1.5	75.16	76.51	71.31	75.56	83.87	85.9	72.26	79.44	75.35	79.14	**77.45**
Mean	**77.97**	**77.84**	**76.55**	**78.04**	**86.25**	**84.73**	**76.14**	**79.11**	**78.25**	**79.48**	
Llama 3											
Temp 0.5	69.75	65.99	64.4	62.91	75.52	70.13	75.58	71.02	71.66	68.63	**69.56**
Temp 1	70.61	67.43	61.83	61.48	82.68	81.87	73.09	76.68	69.73	70.44	**71.59**
Temp 1.5	54.22	59.34	45.1	47.97	60.18	64.24	44.69	47.81	42.28	46.2	**51.2**
Mean	**64.86**	**64.25**	**57.11**	**57.45**	**72.8**	**72.08**	**64.45**	**65.17**	**61.22**	**61.76**	
Mixtral											
Temp 0.5	85.5	83.28	71.04	70.28	87.24	85.61	72.01	74.04	84.32	83.77	**79.71**
Temp 1	85.59	84.67	70.47	69.36	86.75	87.98	71.82	73.83	84.38	86.52	**80.14**
Temp 1.5	83.32	87.96	68.69	70.12	81.22	87.31	72.92	77.75	82.33	86.03	**79.77**
Mean	**84.81**	**85.31**	**70.07**	**69.92**	**85.07**	**86.97**	**72.25**	**75.2**	**83.68**	**85.44**	
DeepSeek											
Temp 0.5	82.32	83.61	74.33	74.32	87.81	85.85	77.35	77.47	79.36	79.09	**80.15**
Temp 1	81.47	82.38	70	73.35	86.74	88.32	75.67	79.49	78.05	79.95	**79.54**
Temp 1.5	68.46	72.9	58.2	61.5	71.43	75.58	61.42	66.69	65.2	69.46	**67.08**
Mean	**77.42**	**79.63**	**67.51**	**69.72**	**81.99**	**83.25**	**71.48**	**74.55**	**74.2**	**76.17**	
oss 120b											
Temp 0.5	80.82	83.81	72.21	76.15	85.27	94.84	83.29	90.46	88.61	94.68	**85.01**
Temp 1	84.41	86.29	70.55	78.27	90.24	95.92	83.85	88.95	87.06	90.33	**85.59**
Temp 1.5	82.88	85.45	76.61	79.94	85.43	93.17	85.91	92.33	87.19	91.15	**86.01**
Mean	**82.7**	**85.18**	**73.12**	**78.12**	**86.98**	**94.64**	**84.35**	**90.58**	**87.62**	**92.05**	
oss 20b											
Temp 0.5	69.73	75.4	64.44	72.52	77.82	84.53	77.22	84.51	82.11	89.17	**77.75**
Temp 1	67.16	72.64	62.46	69.59	73.67	80.74	72.22	80.06	81.02	88.61	**74.82**
Temp 1.5	66.78	71.39	62.5	68.17	71.74	79.79	69.73	76.64	76.8	84.29	**72.78**
Mean	**67.89**	**73.14**	**63.13**	**70.09**	**74.41**	**81.69**	**73.06**	**80.4**	**79.98**	**87.36**	
Grand mean	**77.76**	**79.38**	**67.66**	**70.3**	**82.26**	**84.88**	**73.3**	**77.42**	**79.6**	**82.65**	

Bold in the table represent grand means

## Footnotes

1 This figure does not include items generated for our empirical validation, small-scale AI Anxiety simulation, or expansion of our main simulation to include the *DeepSeek* and *GPT OSS* models.

2 However, when incorporating results from models released after the main simulation, GPT OSS 120b emerged as the top performer, with average final NMIs ranging from 94.57 to 95.35 across temperature settings (see Table 11 in the Appendix). This performance is nearly one percentage point higher than GPT-4o’s best outcome. While further empirical testing is required to validate this finding, these results highlight the potential of open-source models to match (and even slightly surpass) the performance of closed-source counterparts within AI-GENIE.

3 Wang and Wang (2022) called this factor *learning*, but we expanded this term to *learning anxiety* to give the model more clarity.

## Code Availability

All codes and data can be found in a dedicated Open Science Framework Repository here: https://osf.io/zcytb/
